# Supplementary material for: Menin inhibition impairs metastatic colonization of Ewing sarcoma
Source: JCI Insight. 2026 Sep 1;11(19):e202419. doi: 10.1172/jci.insight.202419 (PMC13726427; doi:10.1172/jci.insight.202419)
Supplement: Supplementary file 2 [file jciinsight-11-202419-s064.pdf]

Menin inhibition impairs metastatic colonization of Ewing sarcoma

Katherine A. Braun, Nicolas M. Garcia, Mohamed A. Ahmed, Darleen S. Tu, Stephanie I. Walter, Emma D. Wrenn, Megan E.B. Dean, Neerja Katiyar, Elizabeth R. Lawlor

JCI Insight unedited blot and gel images

Full unedited blot for Figure 1C – A673

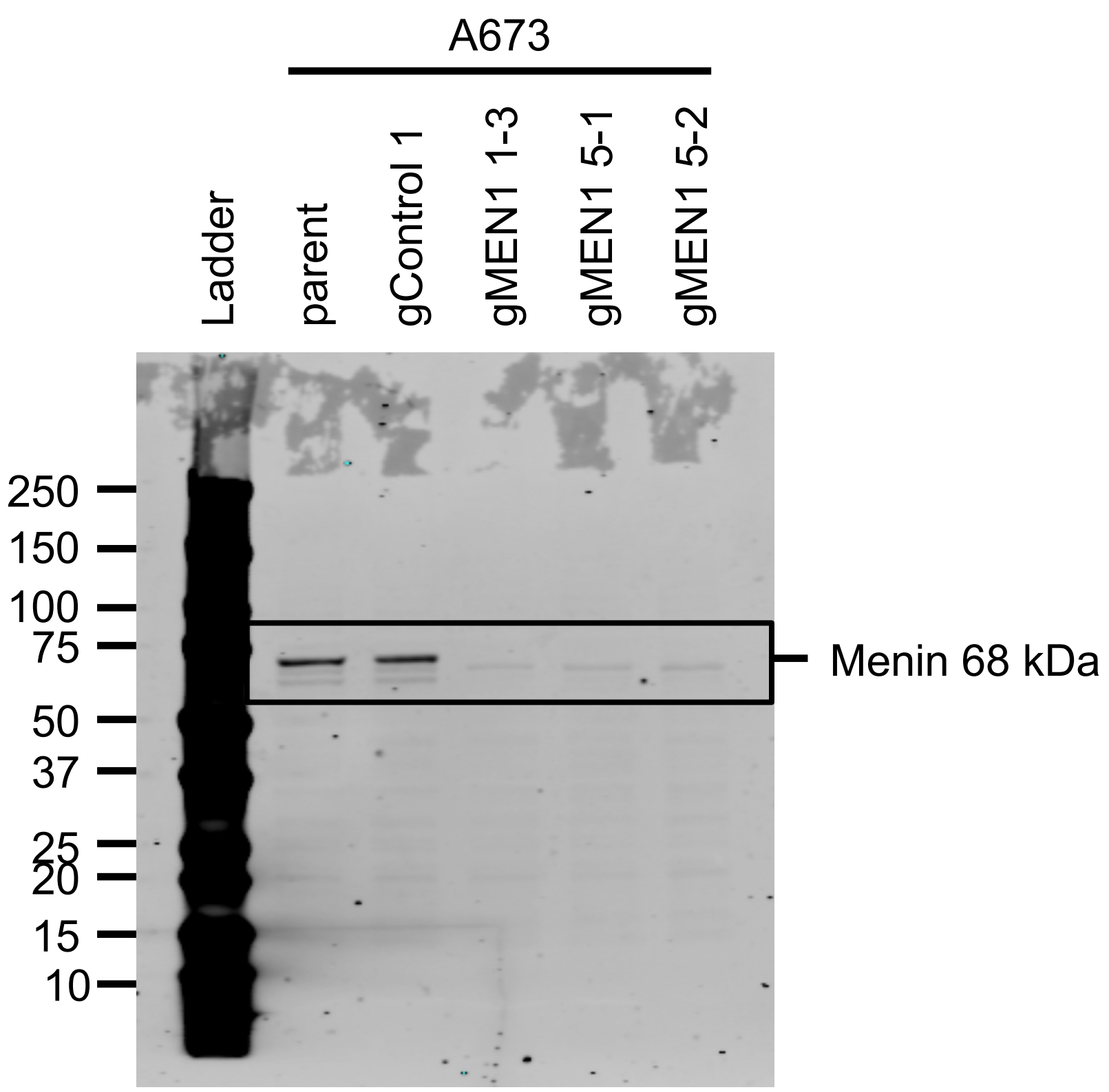

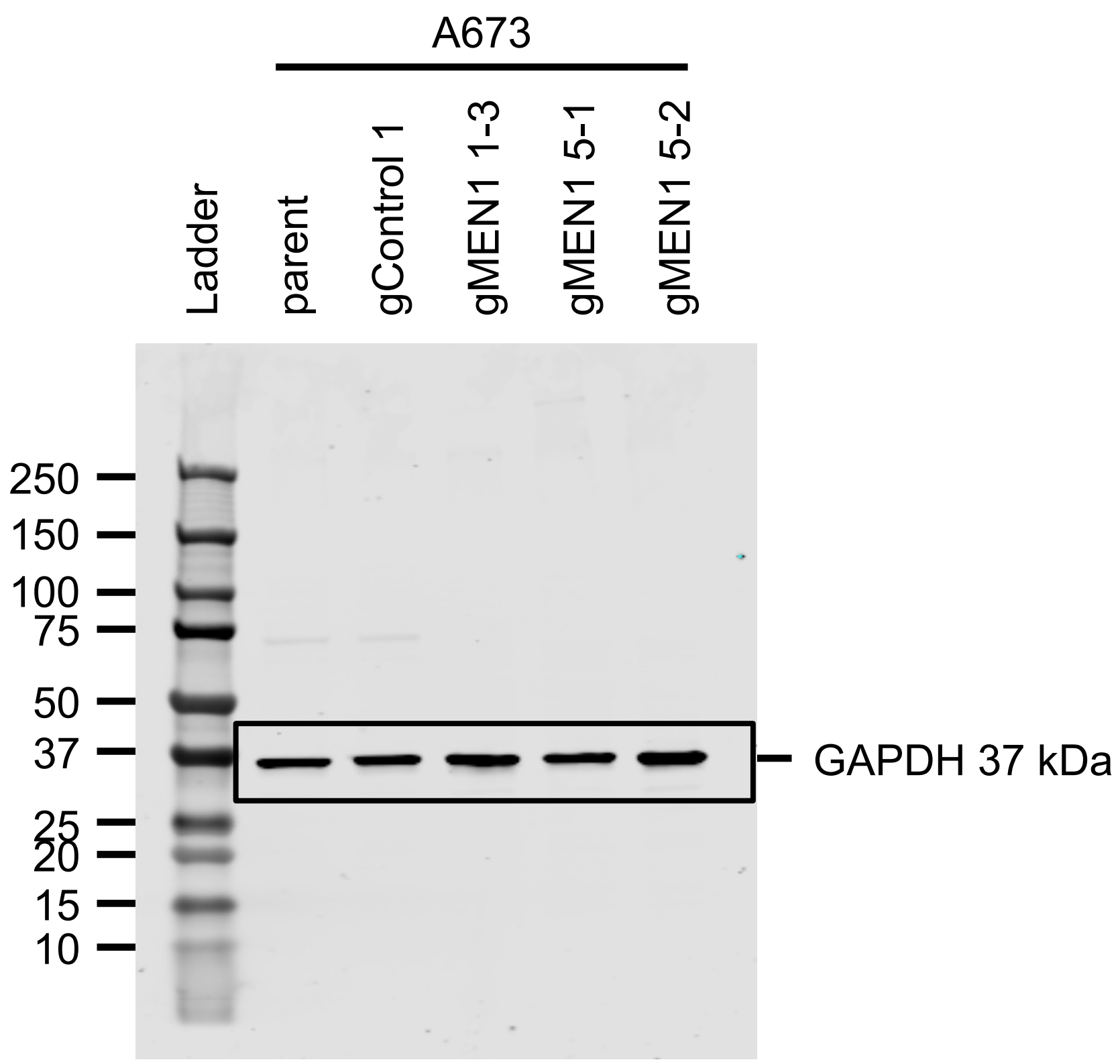

Probed sequentially – α-Menin -> α-GAPDH

Full unedited blot for Figure 1C – A673

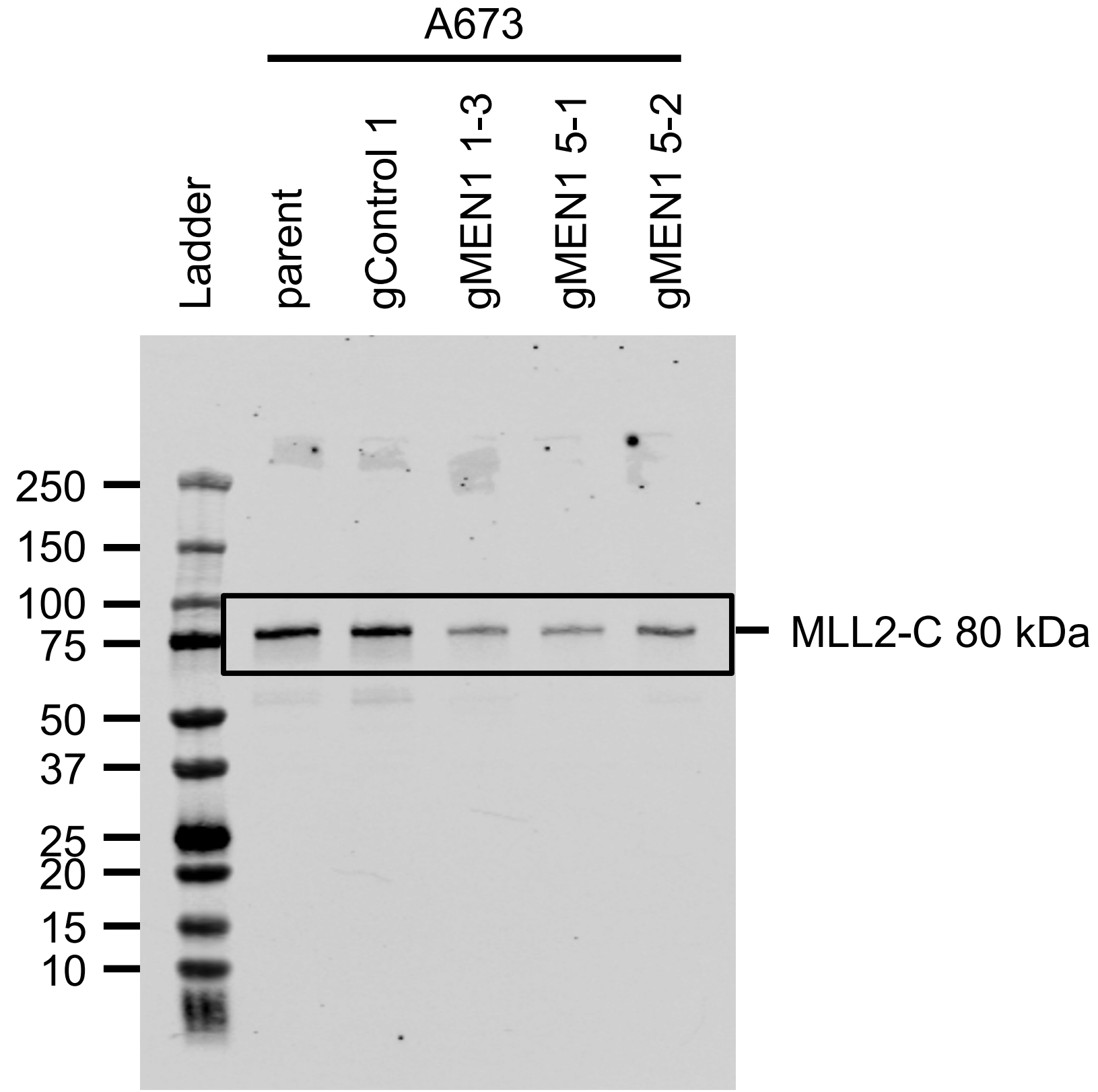

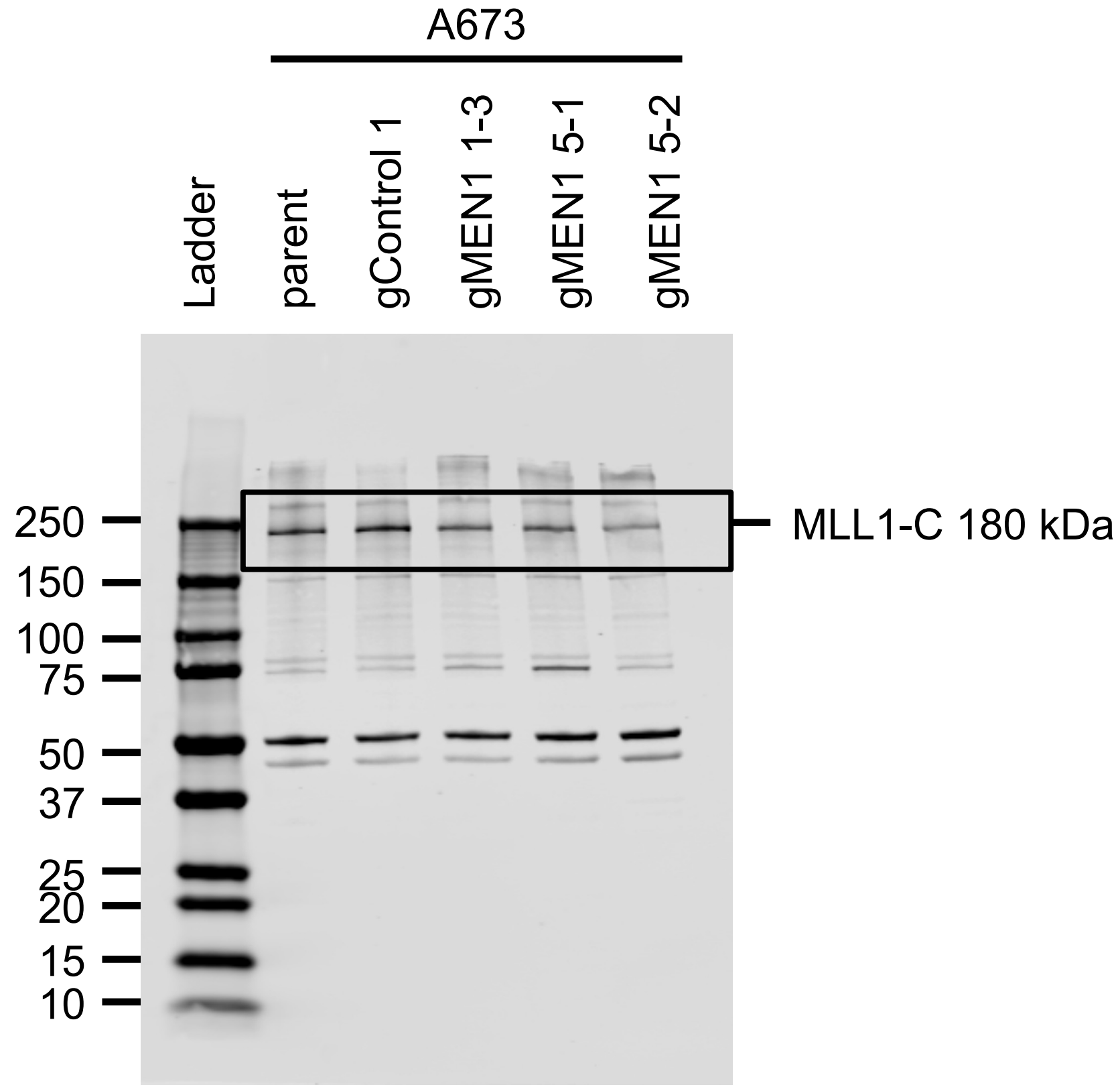

Two separate blots

Full unedited blot for Figure 1C – TC32

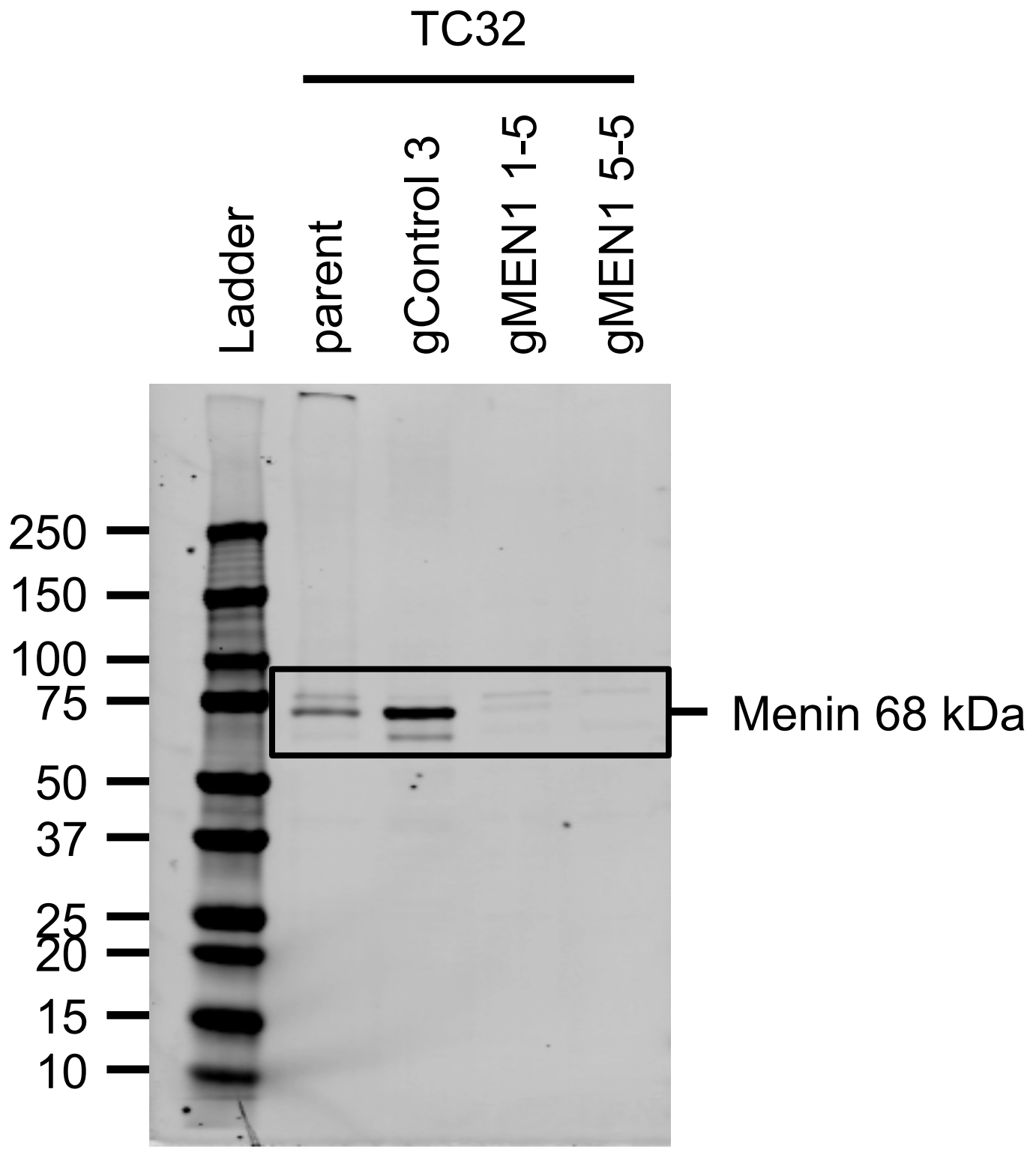

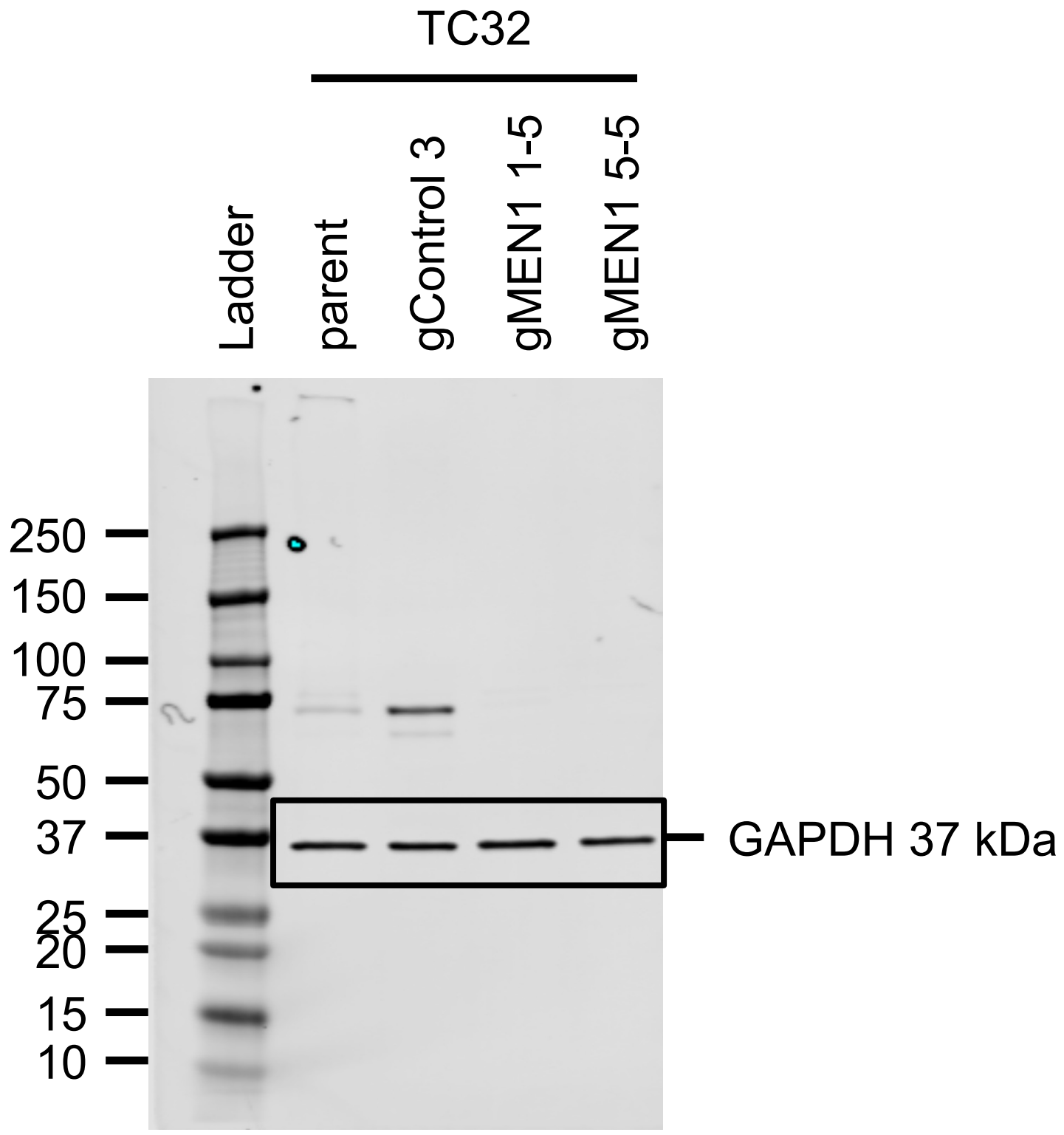

Probed sequentially – α-Menin -> α-GAPDH

Full unedited blot for Figure 1C – TC32

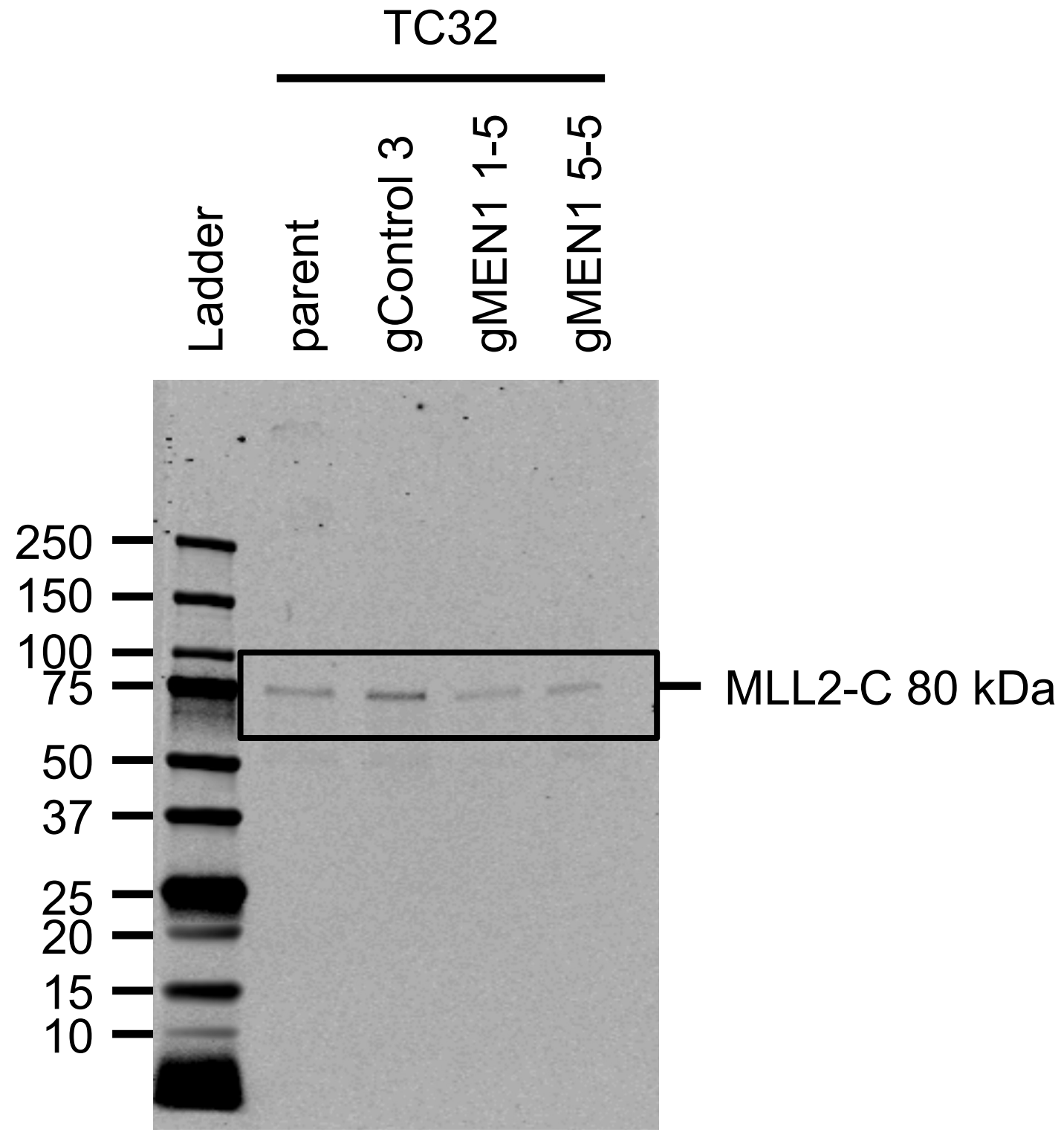

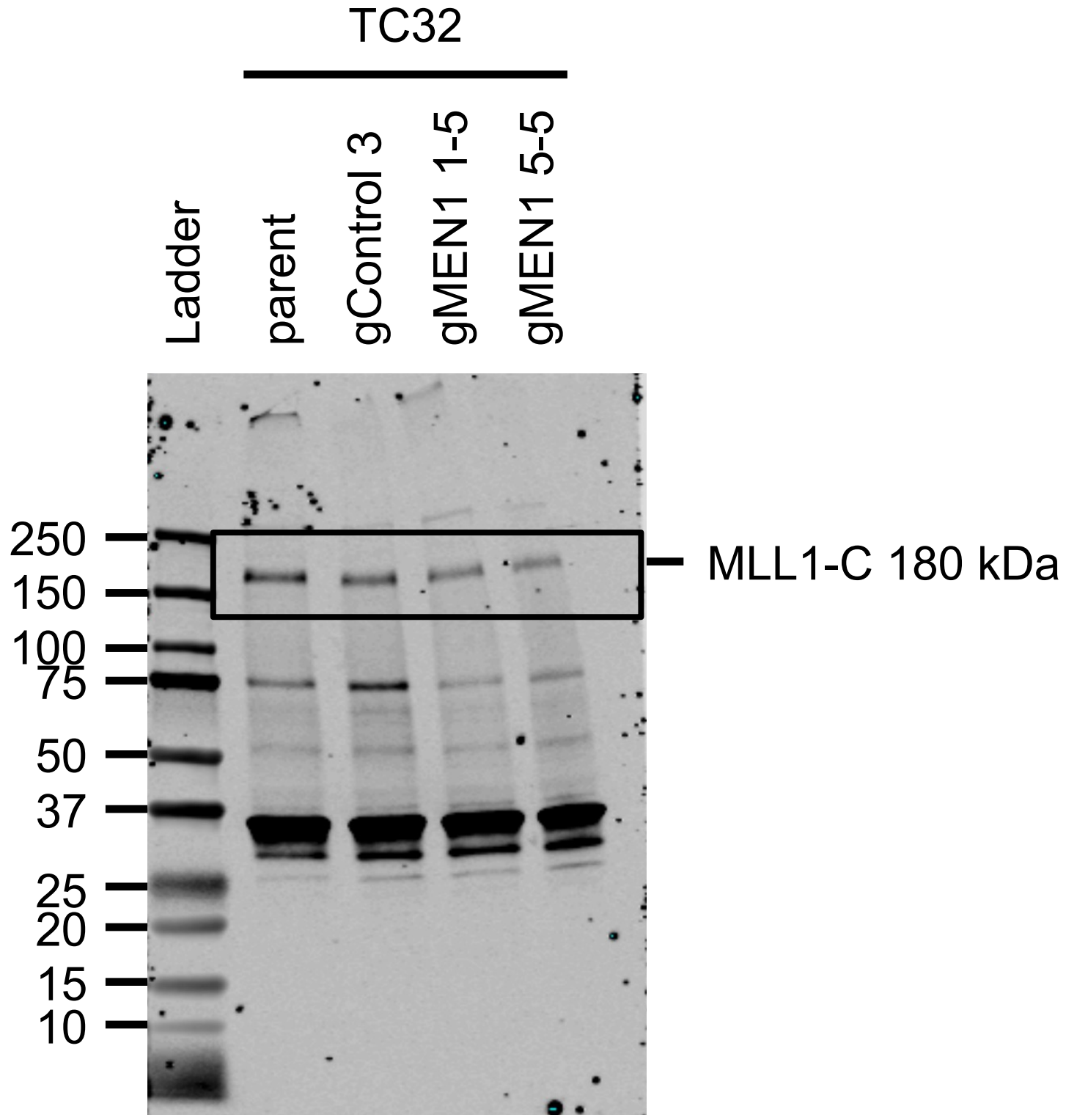

Probed sequentially – α-MLL2-C -> α-MLL1-C

Full unedited blot for Figure 5C

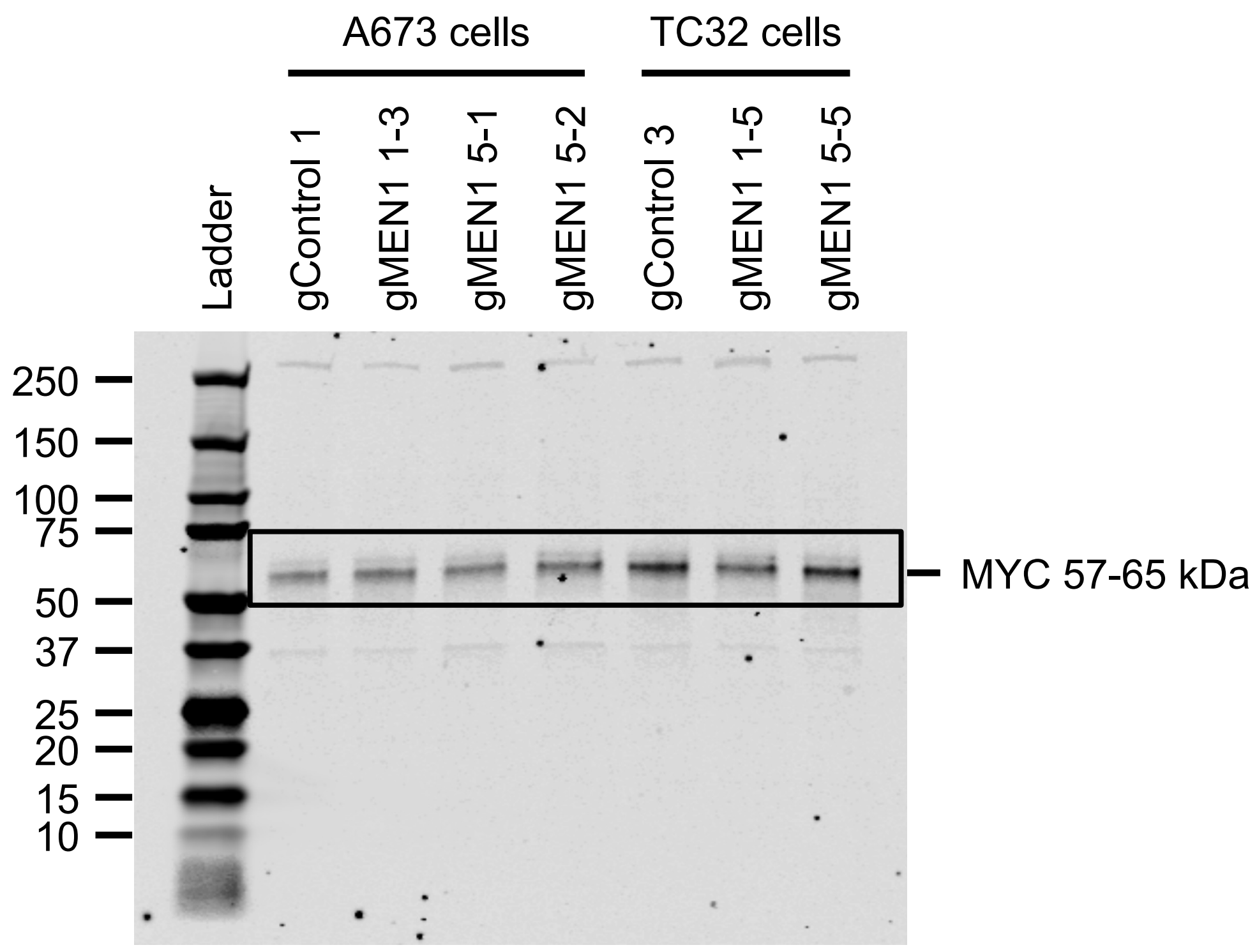

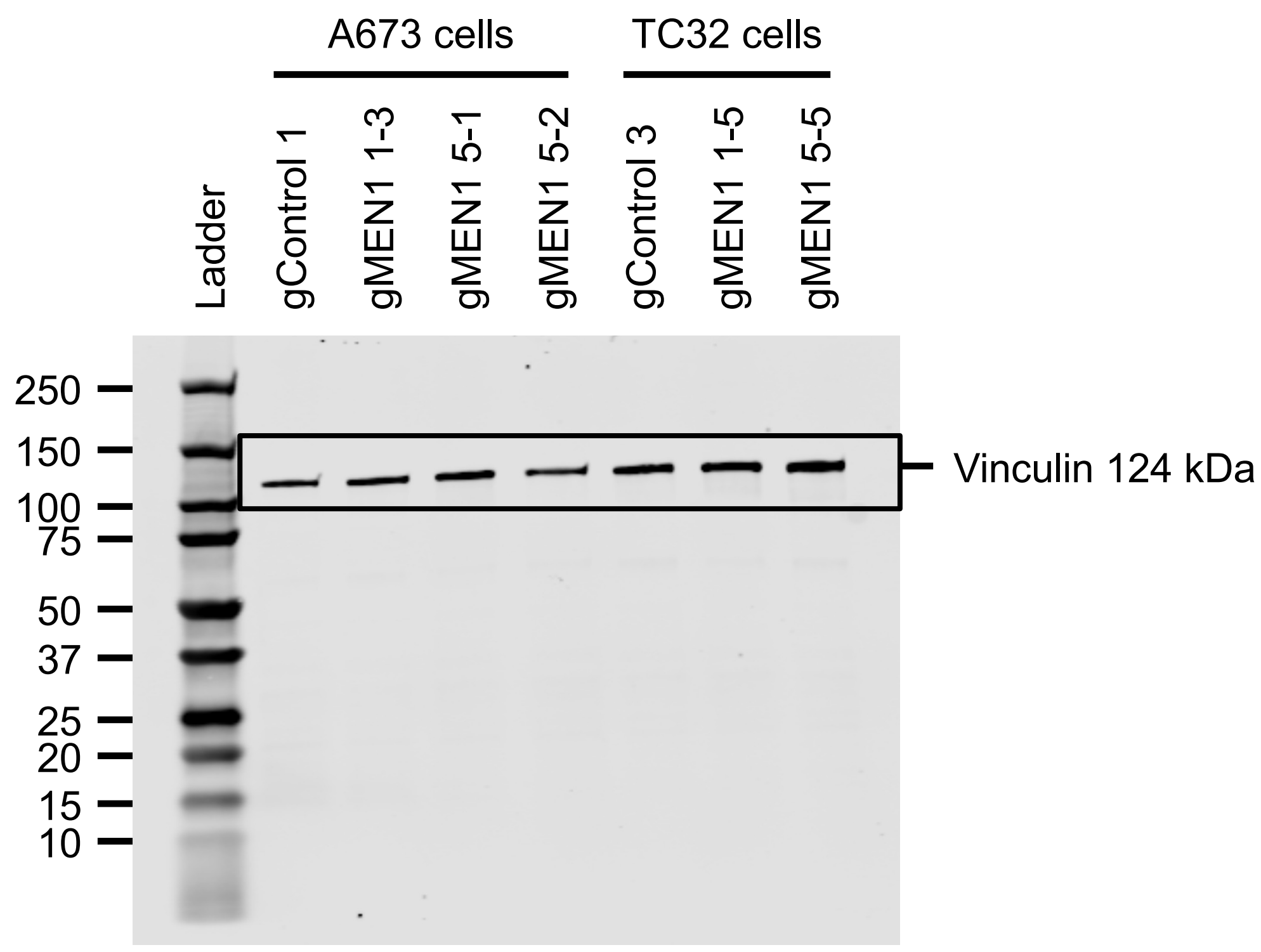

Probed sequentially – α-MYC -> α-Vinculin

Full unedited blot for Figure 5D

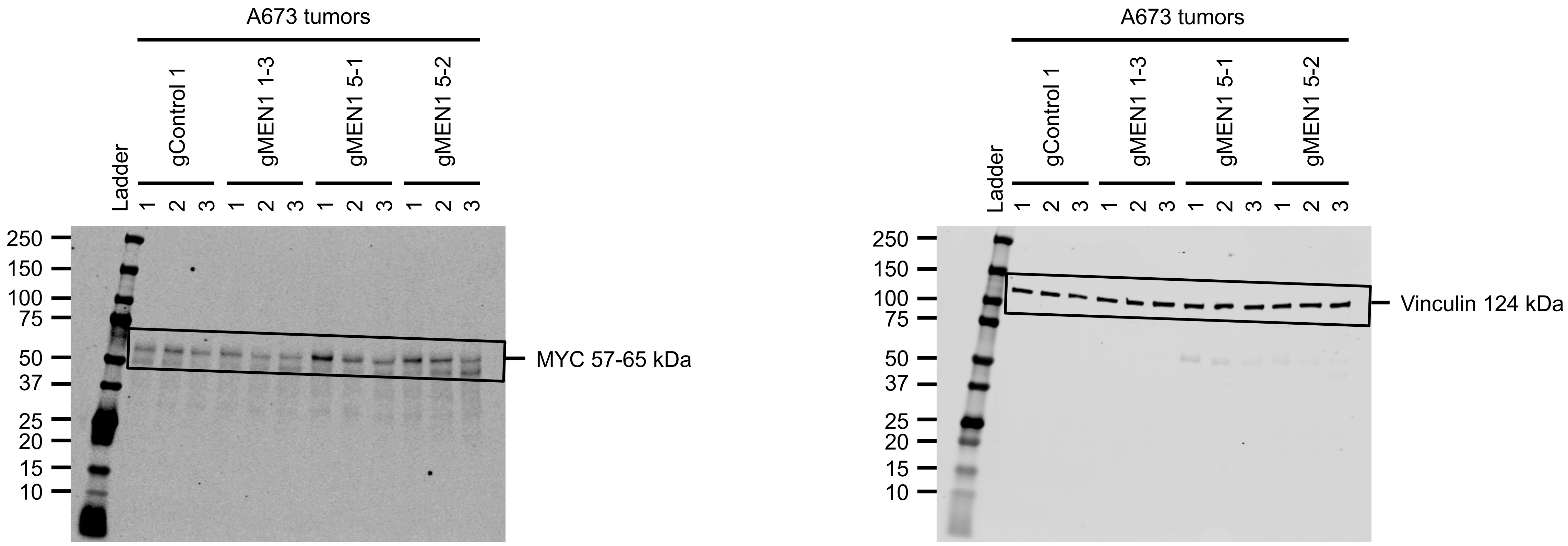

Probed sequentially – α-MYC -> α-Vinculin

Full unedited blot for Figure 5E – A673

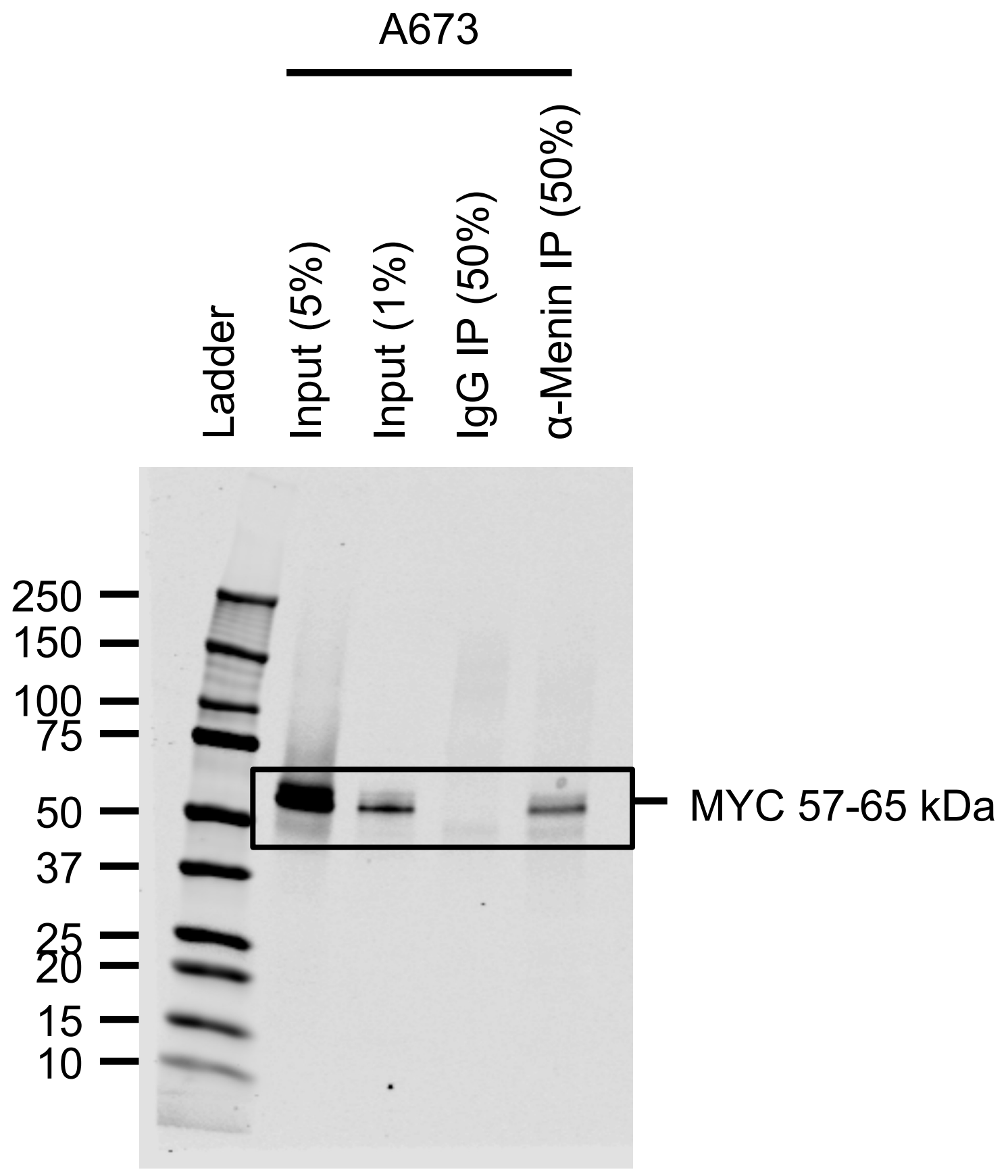

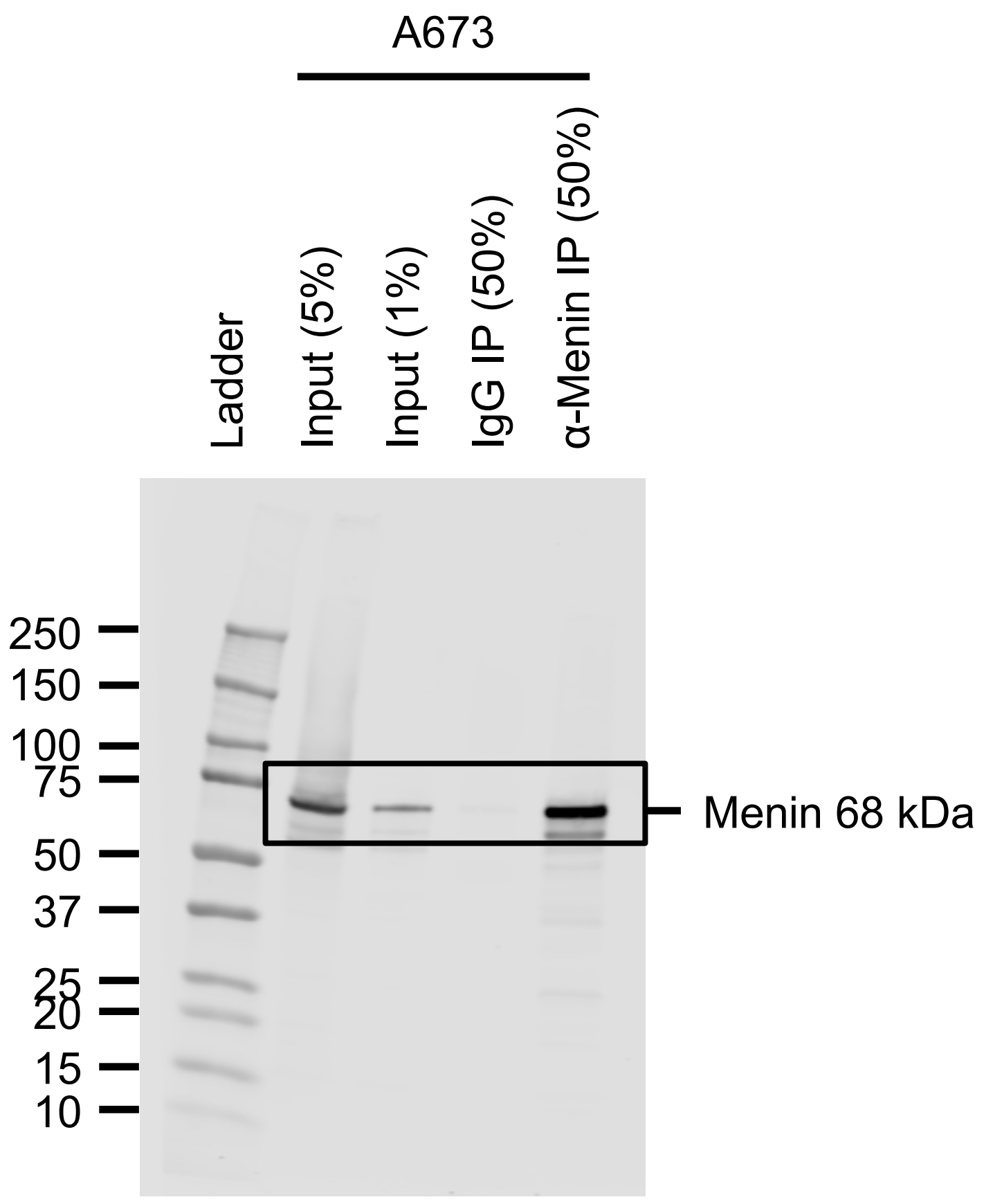

Probed sequentially – α-MYC -> α-Menin

Full unedited blot for Figure 5E – A673

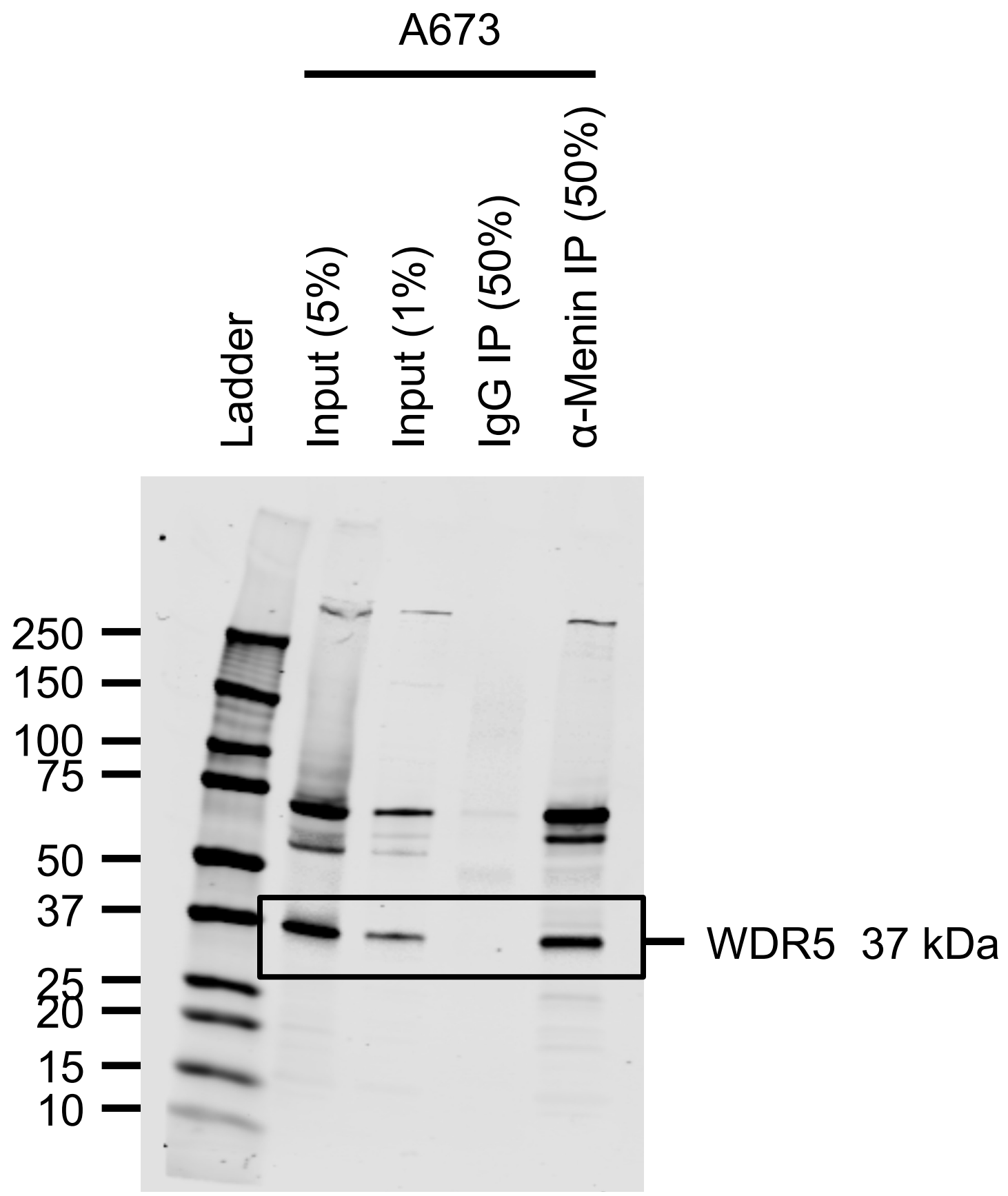

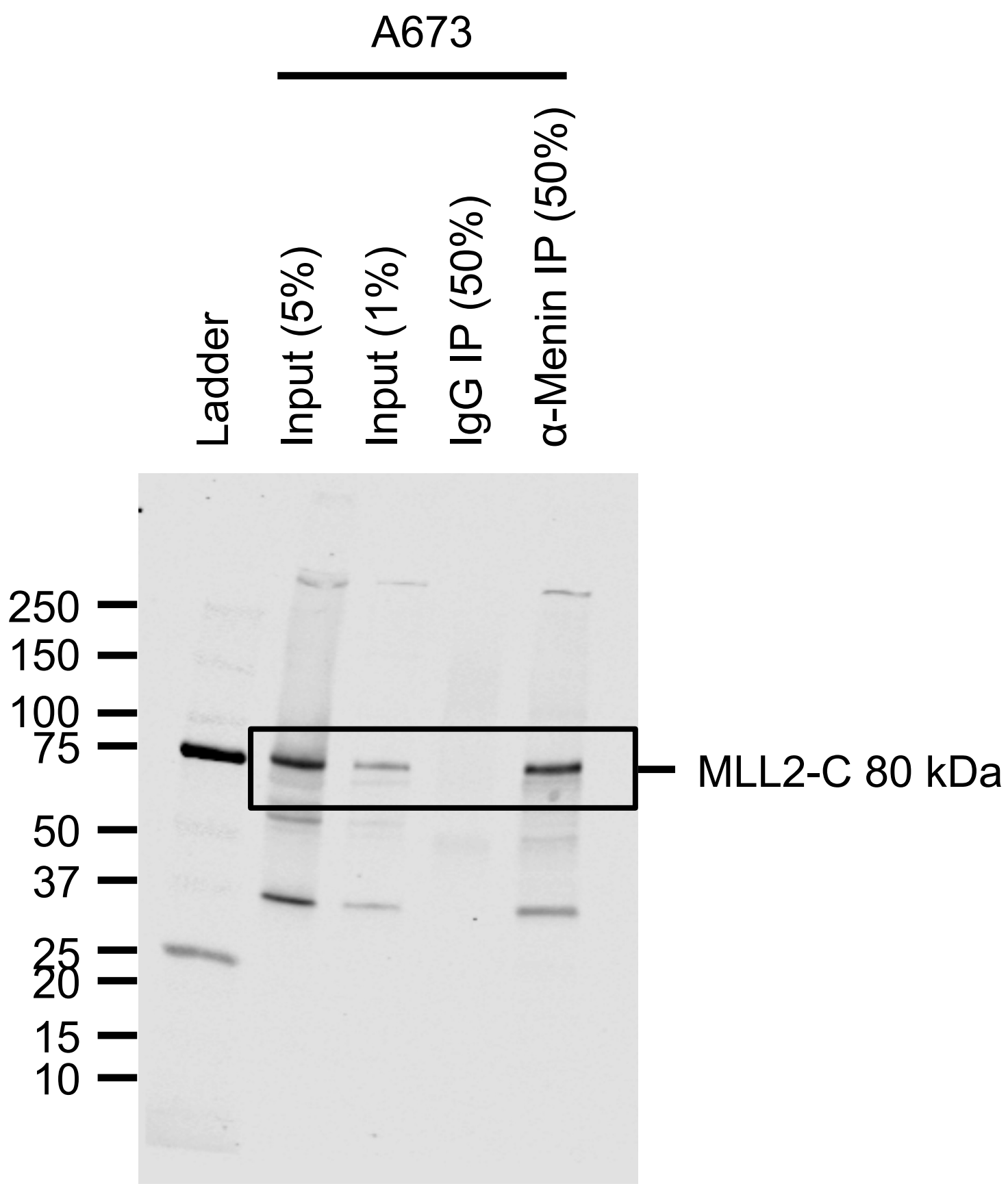

Probed sequentially – α-MYC -> α-Menin -> α-WDR5 -> α-MLL2-C

Full unedited blot for Figure 5E – CHLA10 & U2OS

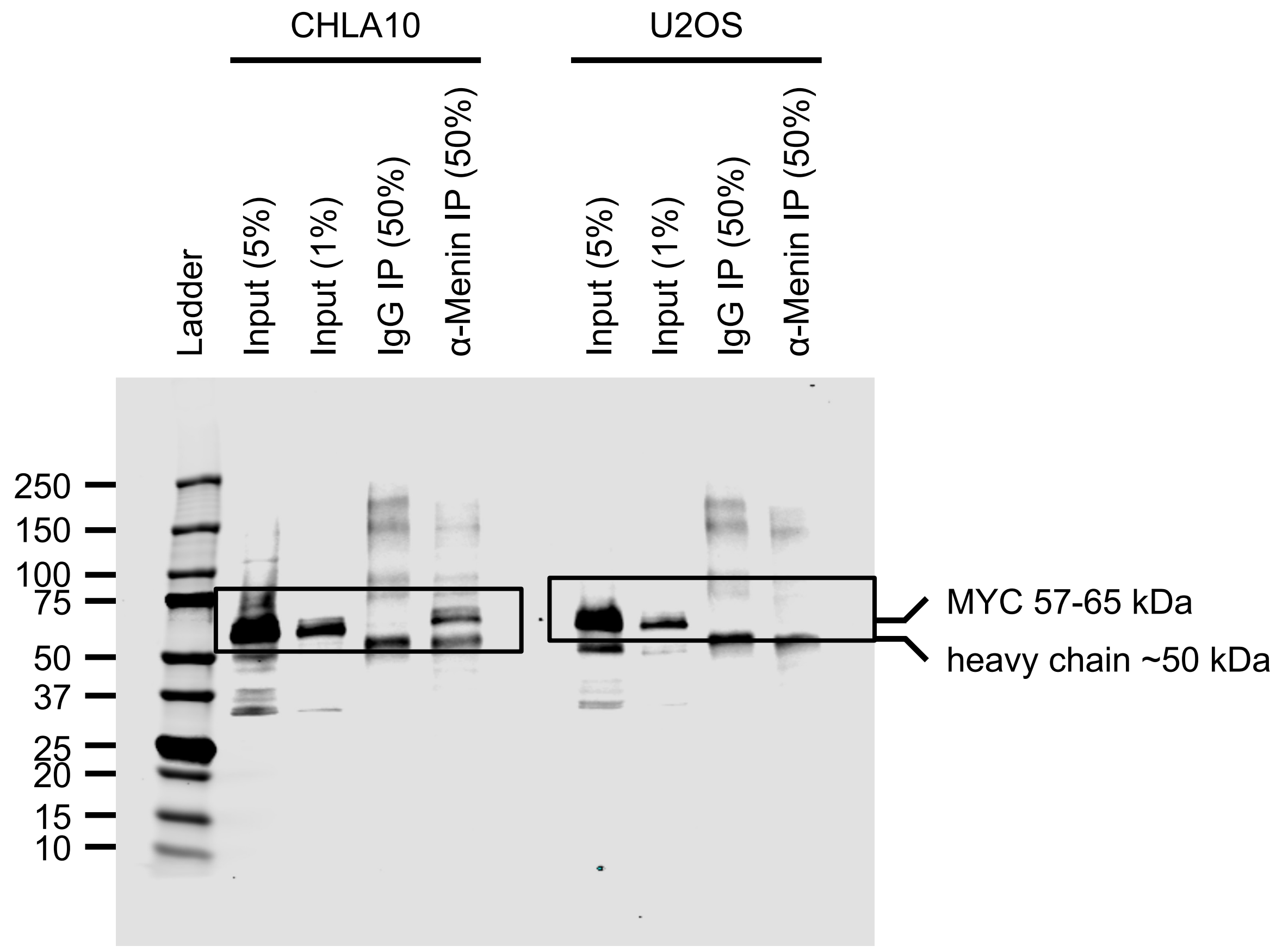

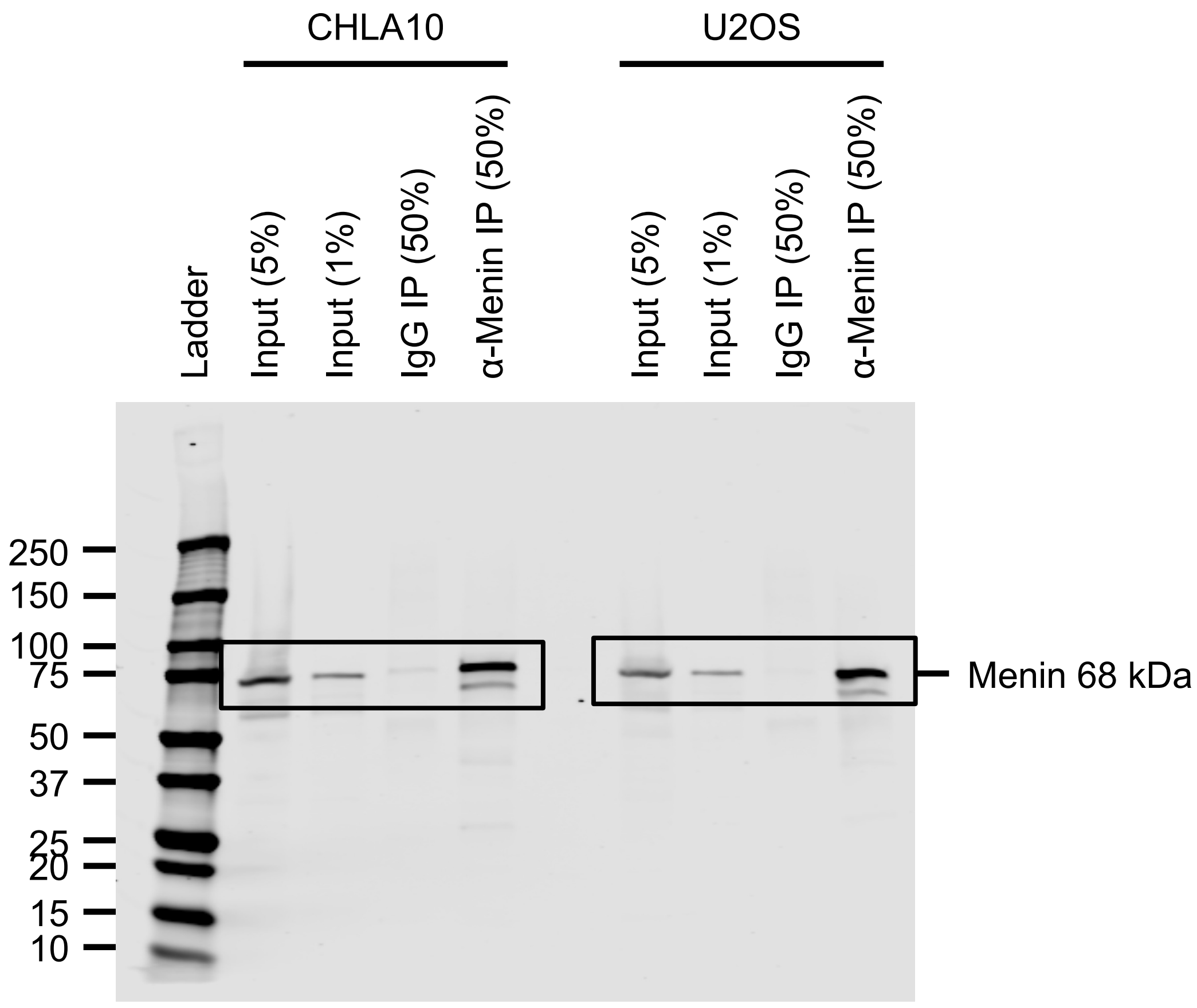

Probed sequentially – α-MYC -> α-Menin

Full unedited blot for Figure 5E – CHLA10 & U2OS

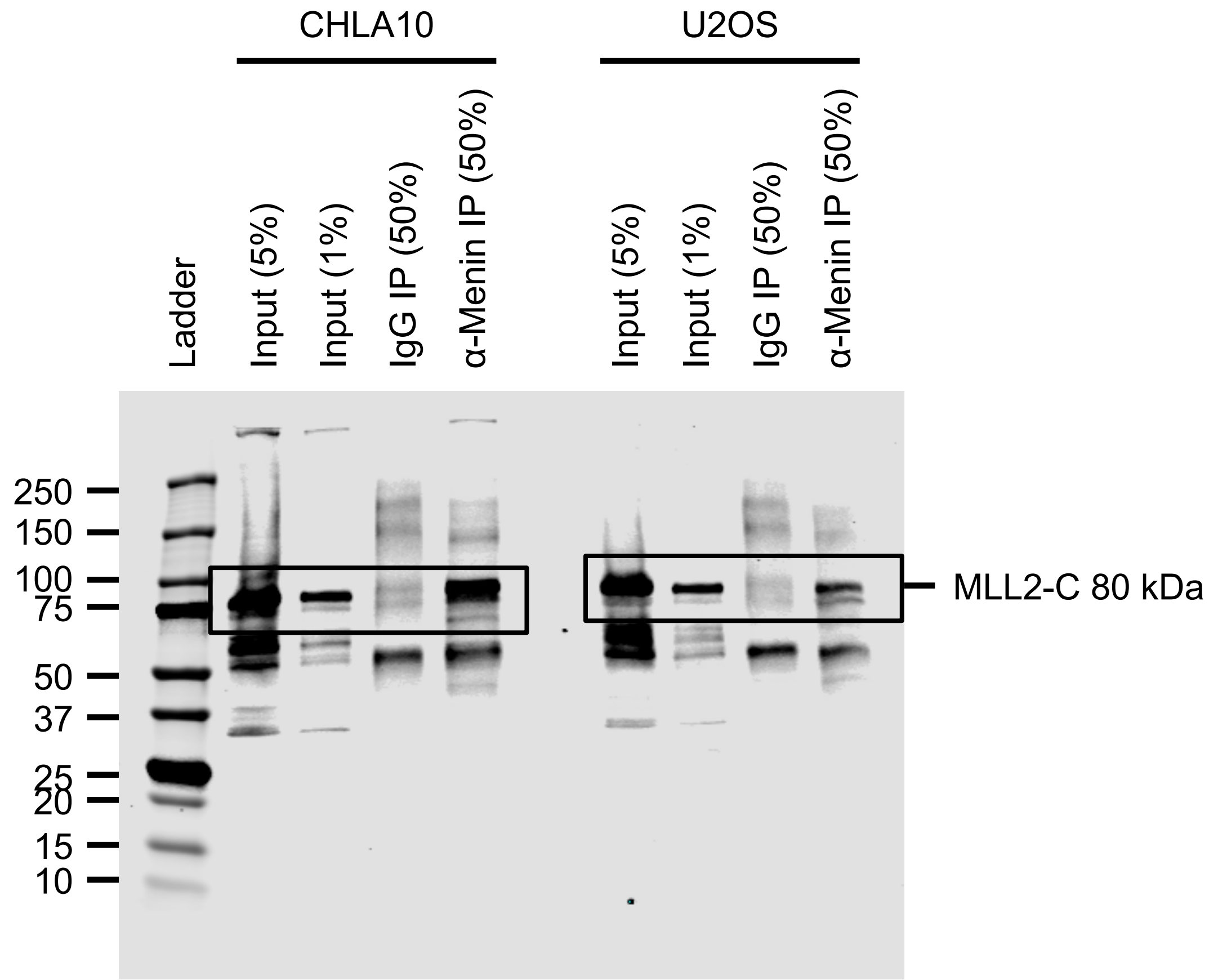

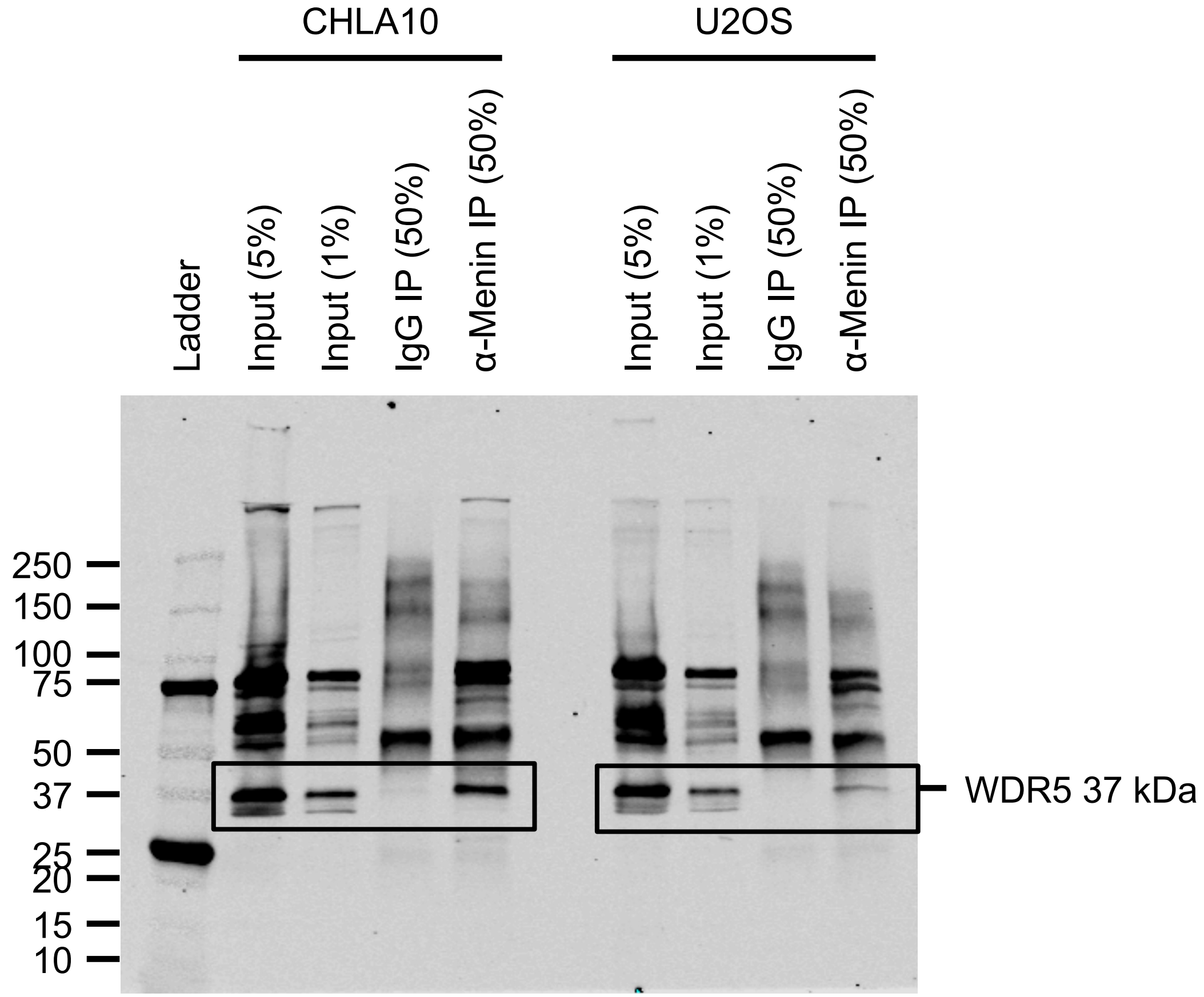

Probed sequentially – α-MYC -> α-Menin -> α-MLL2-C -> α-WDR5

Full unedited blot for Figure 5F – A673 and CHLA10

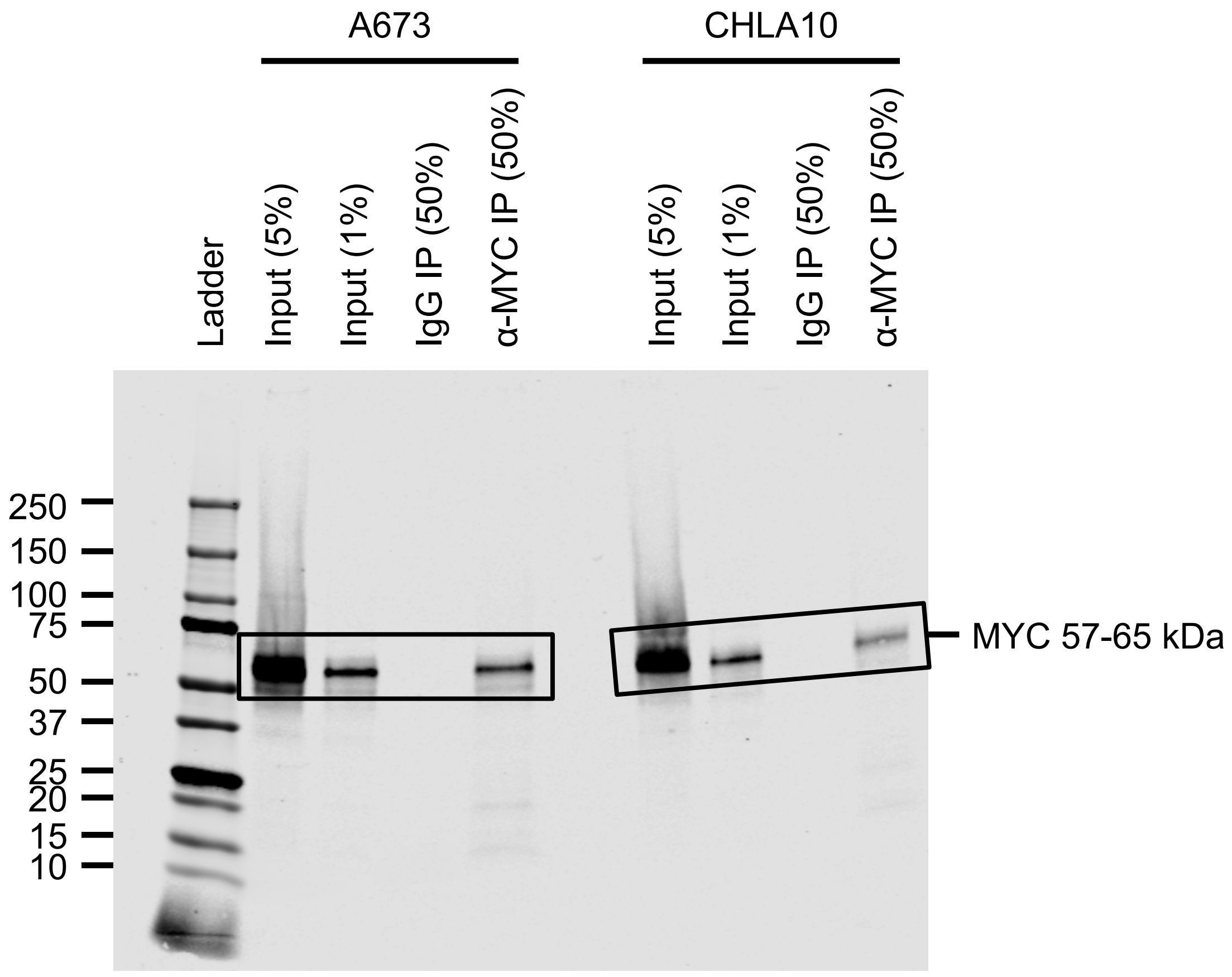

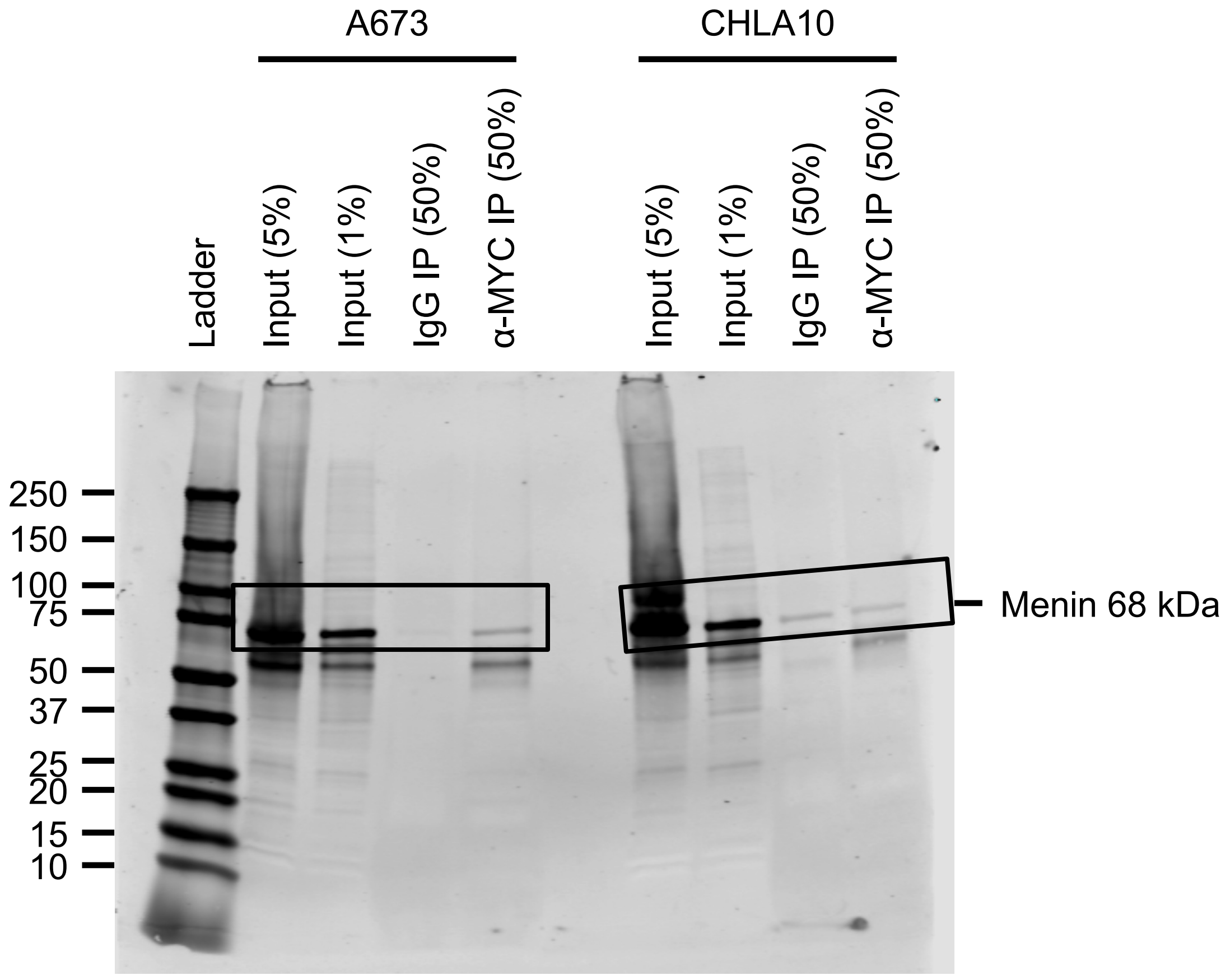

Probed sequentially – α-MYC -> α-Menin

Full unedited blot for Figure 5F – A673 and CHLA10

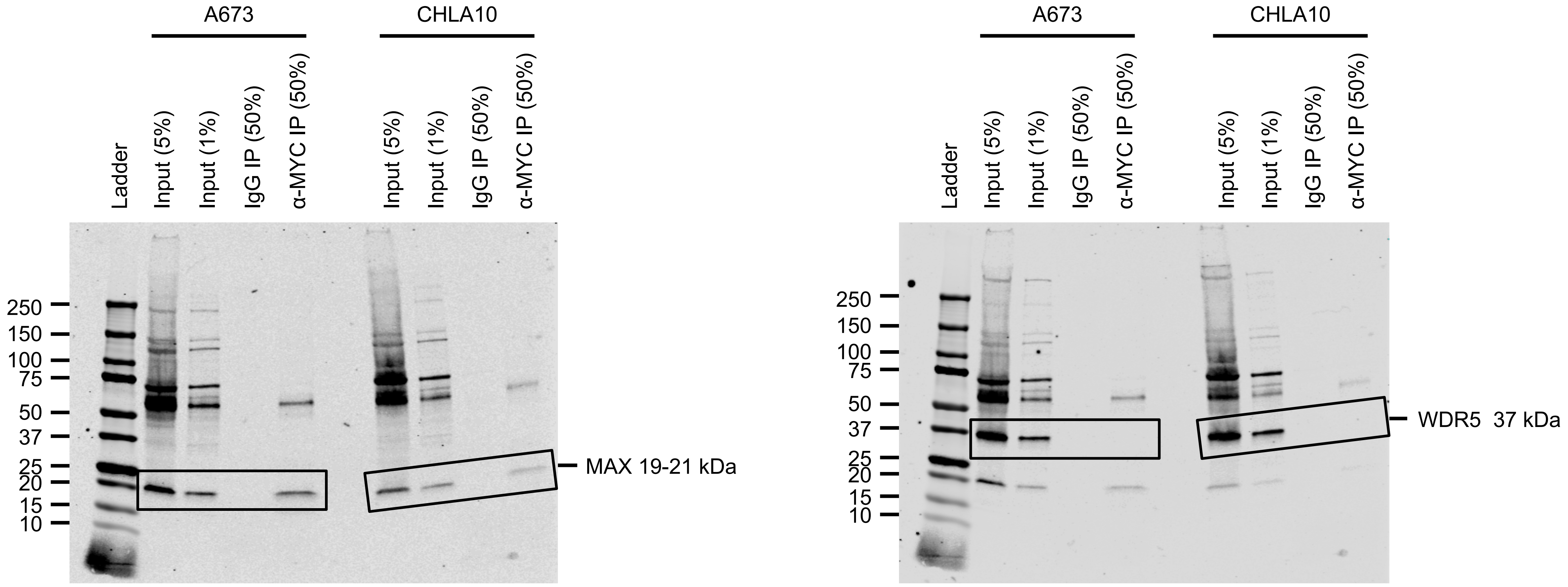

Probed sequentially – α-MYC -> α-Menin -> α-MAX -> α-WDR5

Full unedited blot for Figure 5F – A673 and CHLA10

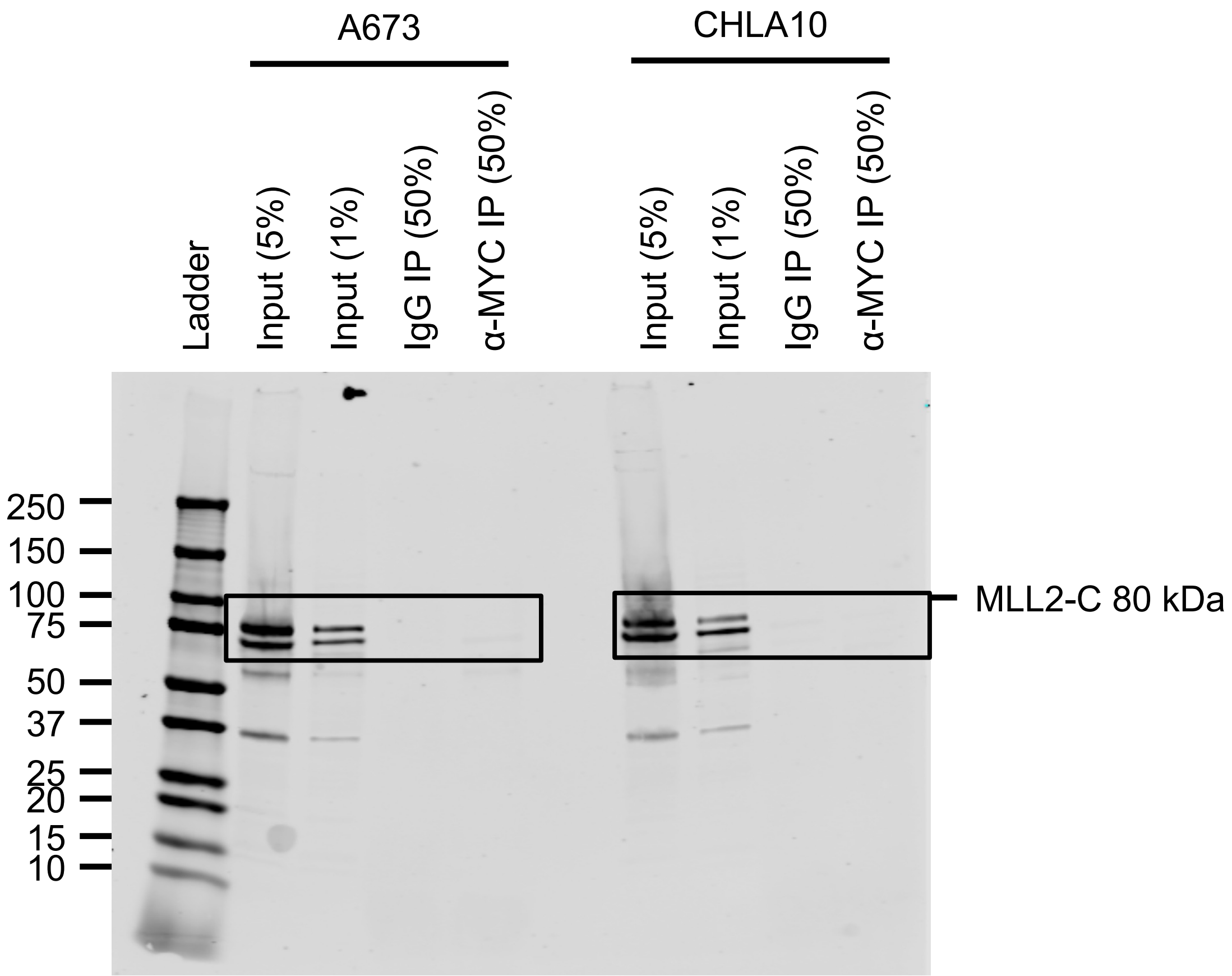

Probed sequentially – α-MYC -> α-Menin -> α-MAX -> α-WDR5 -> MLL2-C

Full unedited blot for Figure 6F

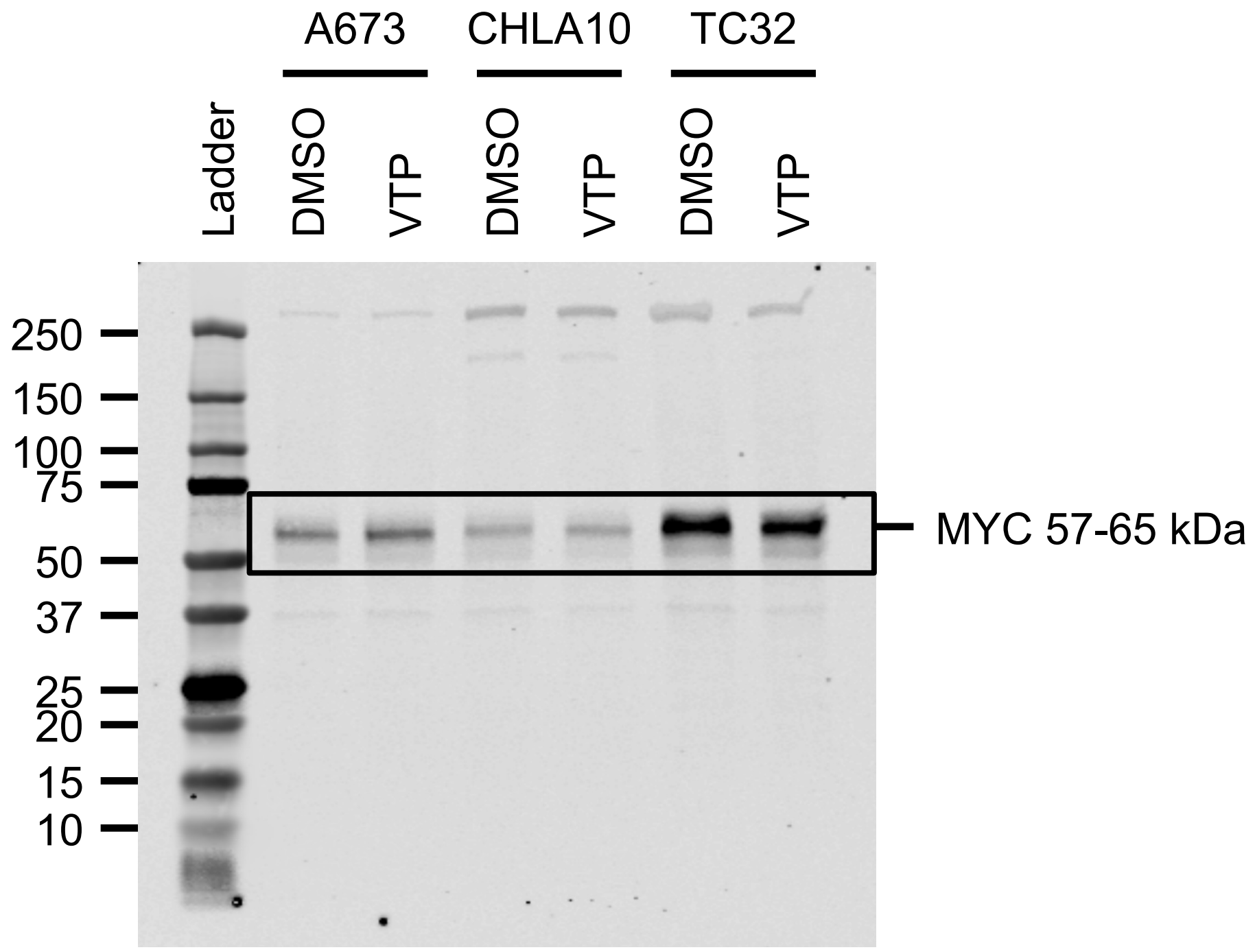

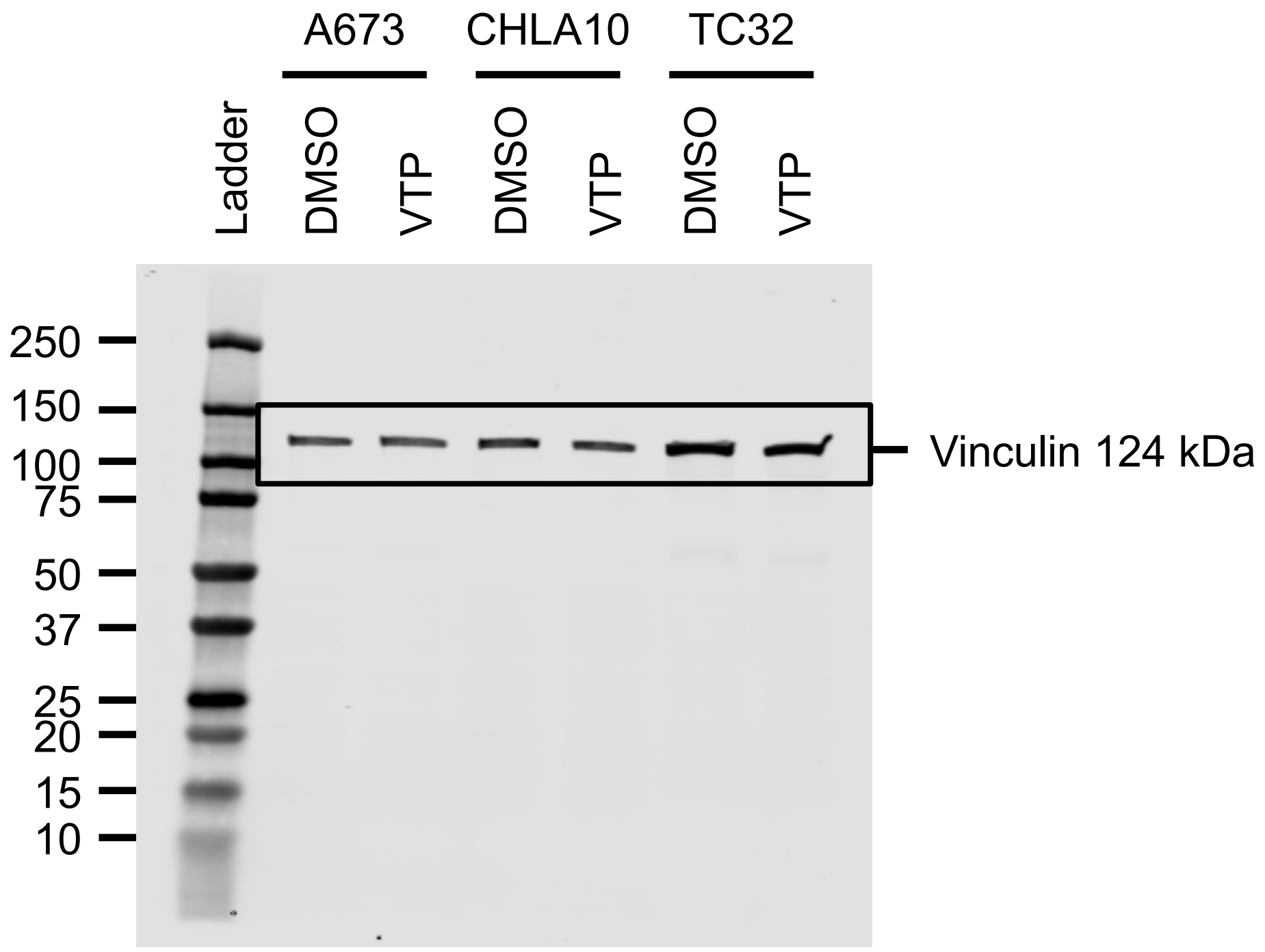

Probed sequentially – α-MYC -> α-Vinculin

Full unedited blot for Figure 6G – A673

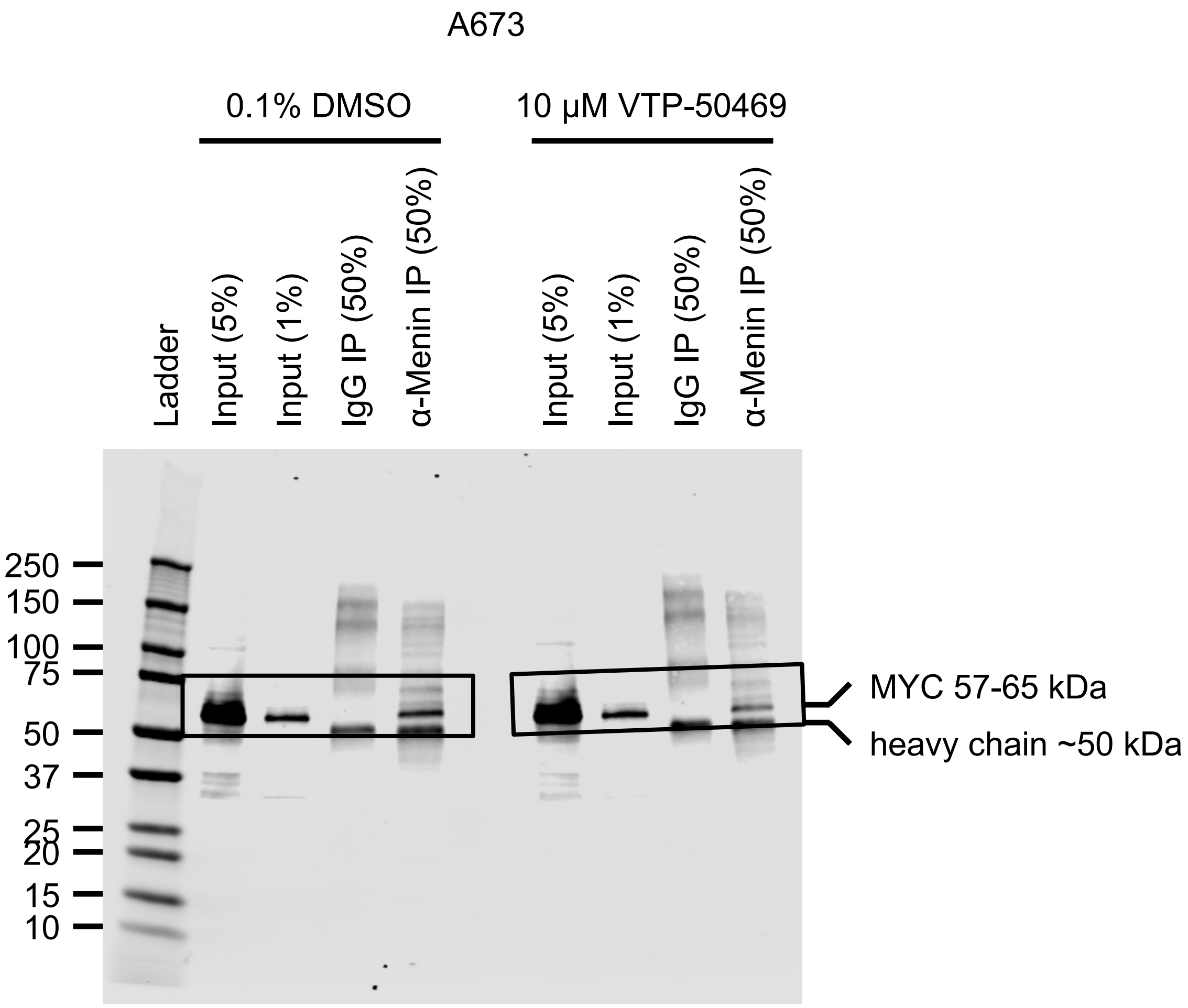

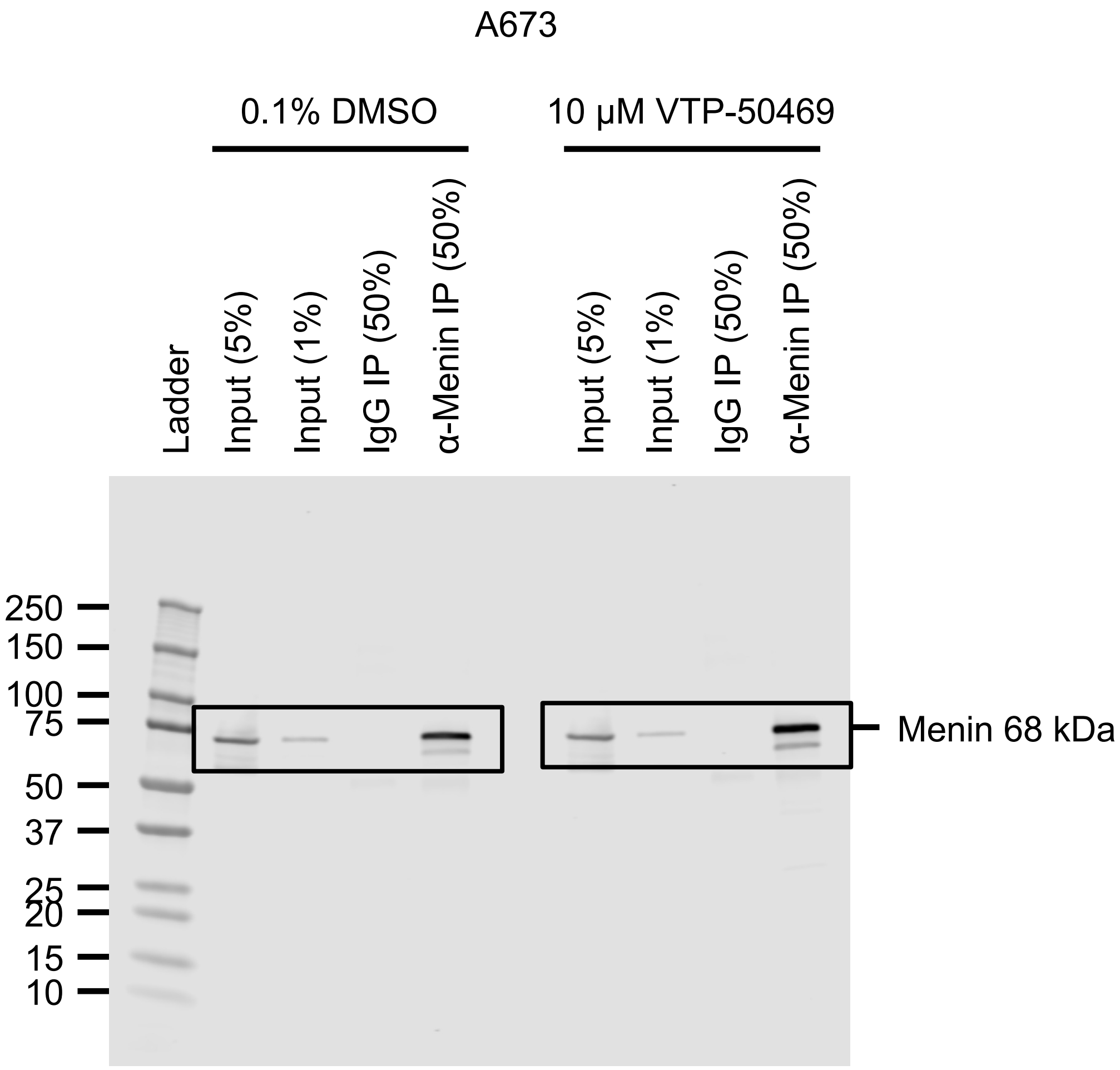

Probed sequentially – α-MYC -> α-Menin

Full unedited blot for Figure 6G – A673

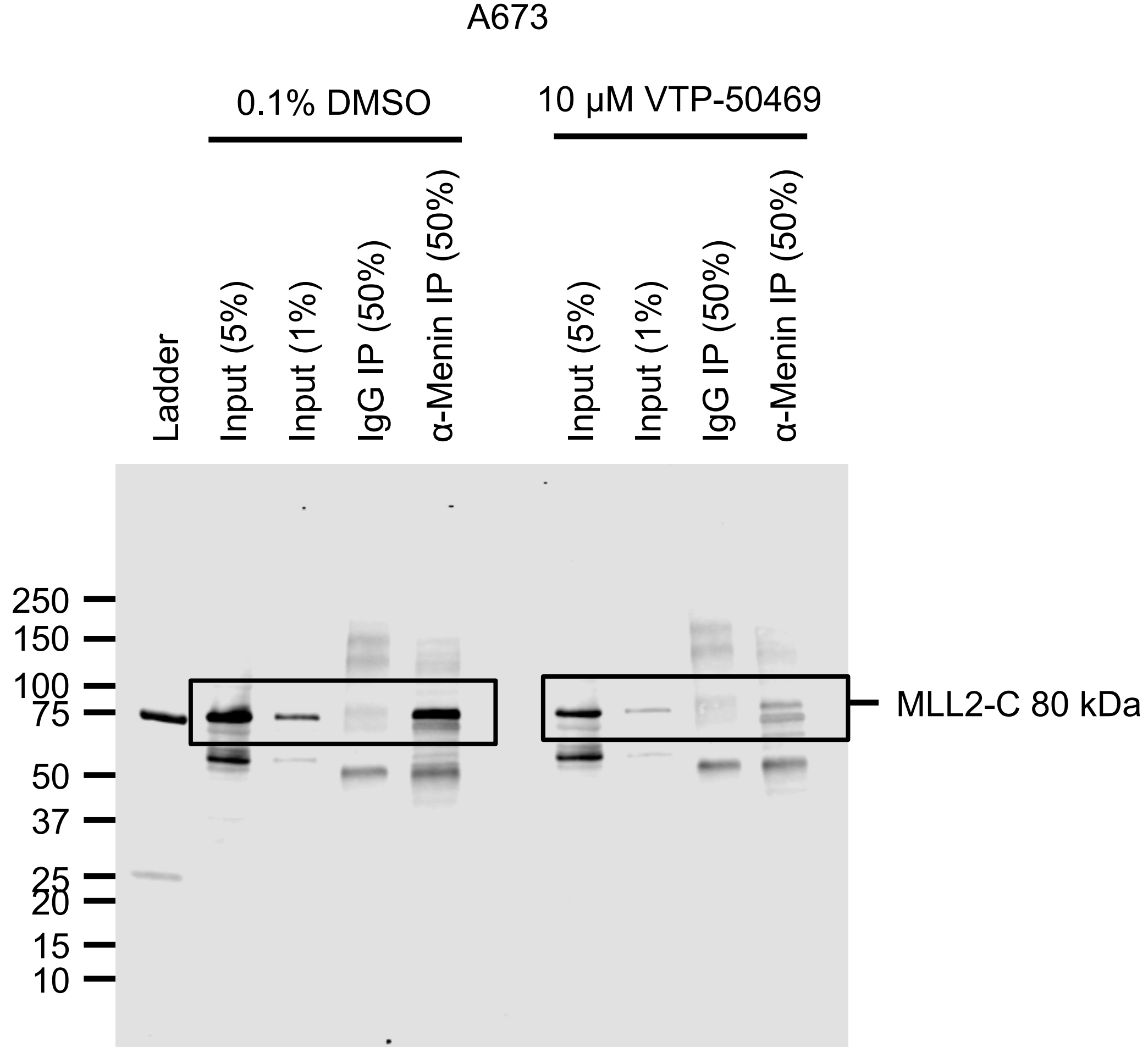

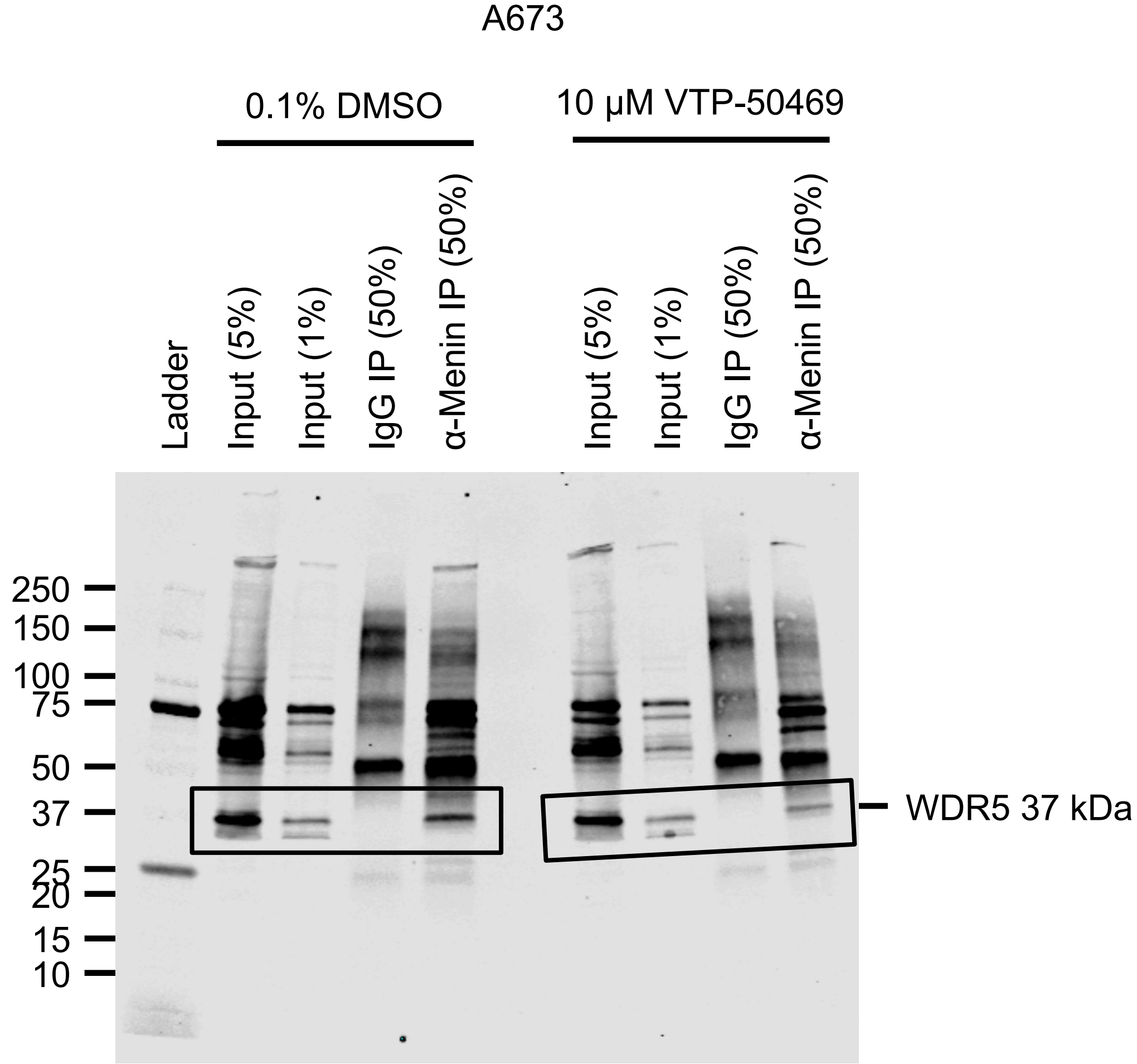

Probed sequentially – α-MYC -> α-Menin -> α-MLL2-C -> α-WDR5

Full unedited blot for Figure 6G – CHLA10

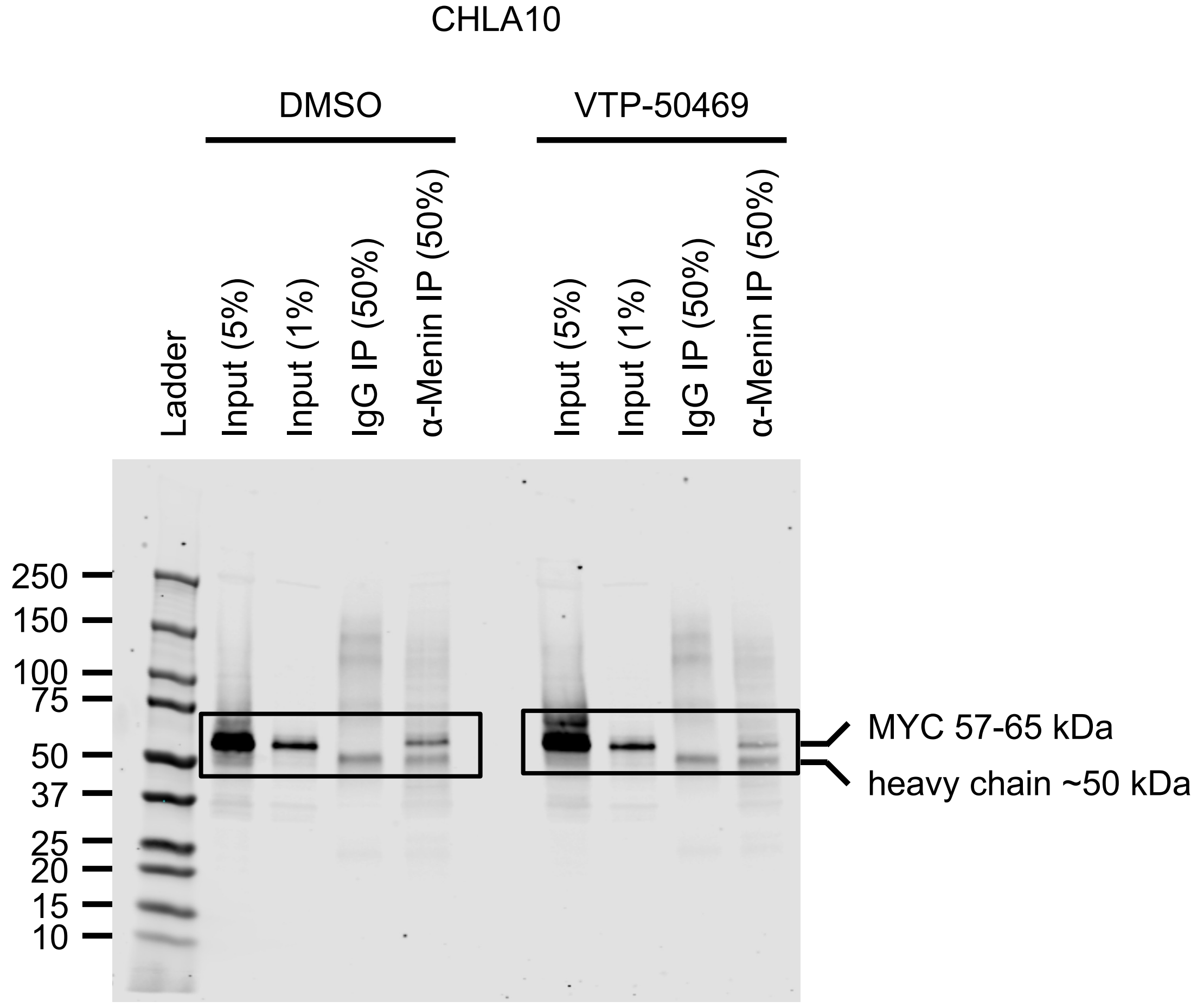

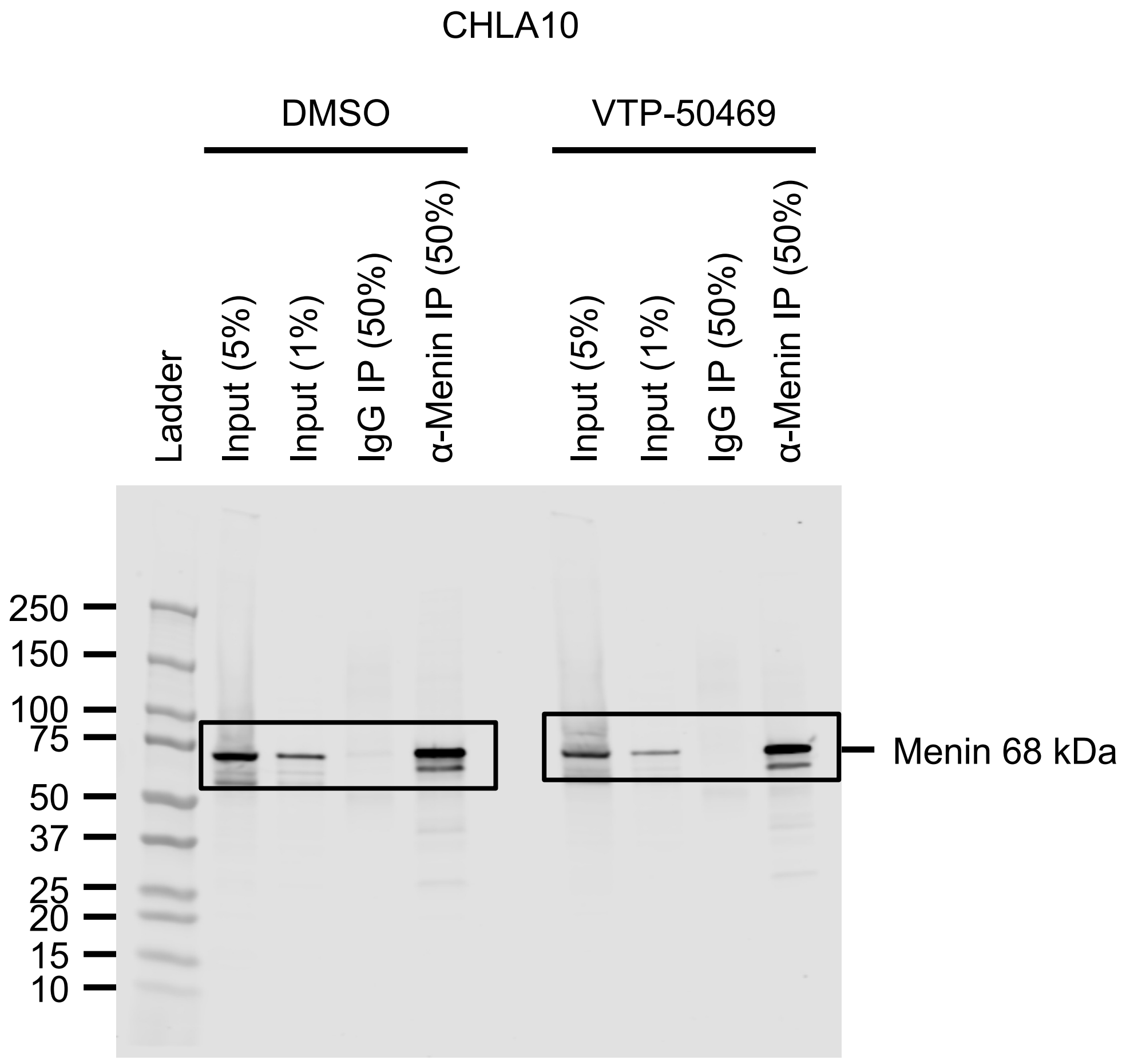

Probed sequentially – α-MYC -> α-Menin

Full unedited blot for Figure 6G – CHLA10

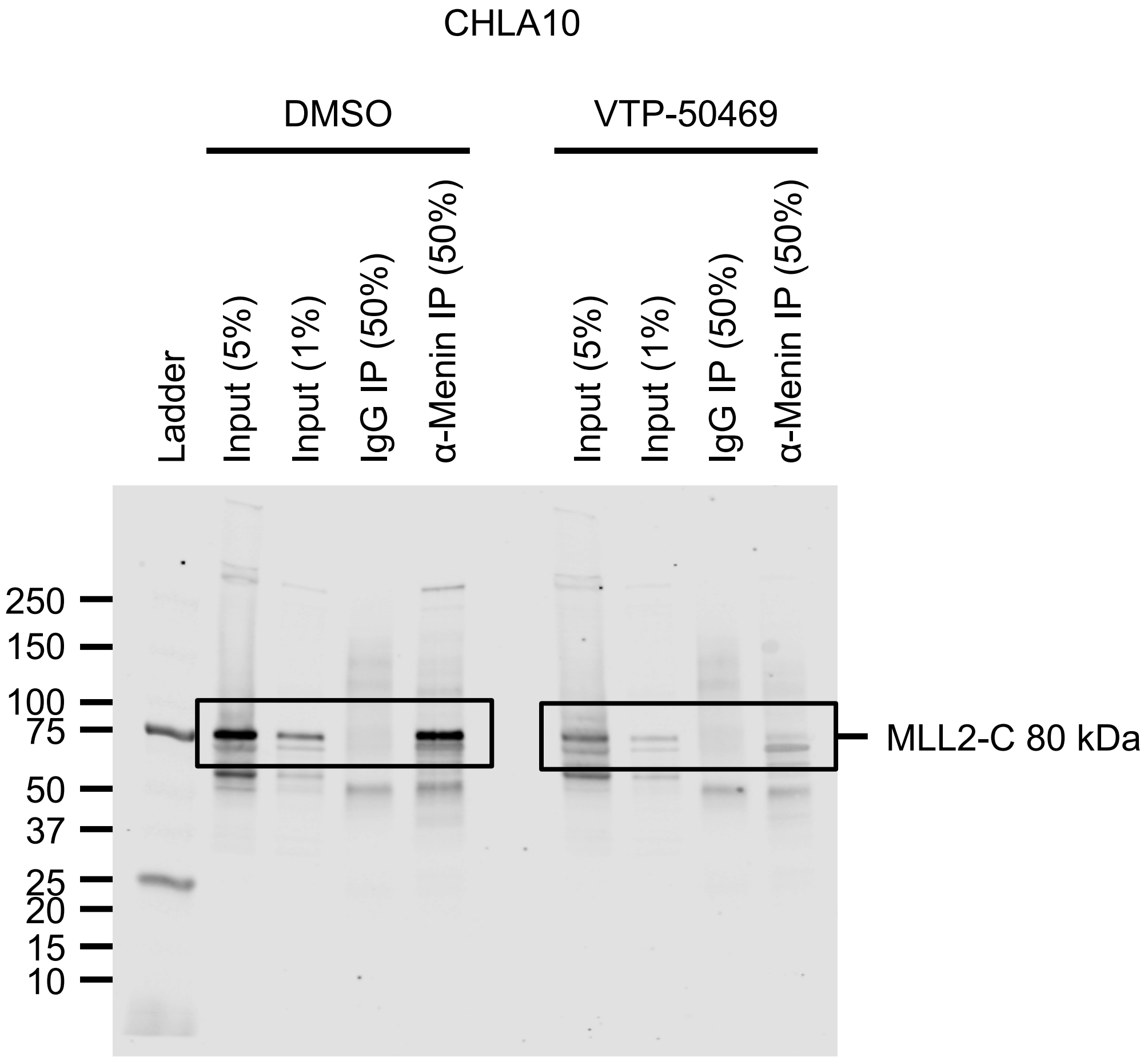

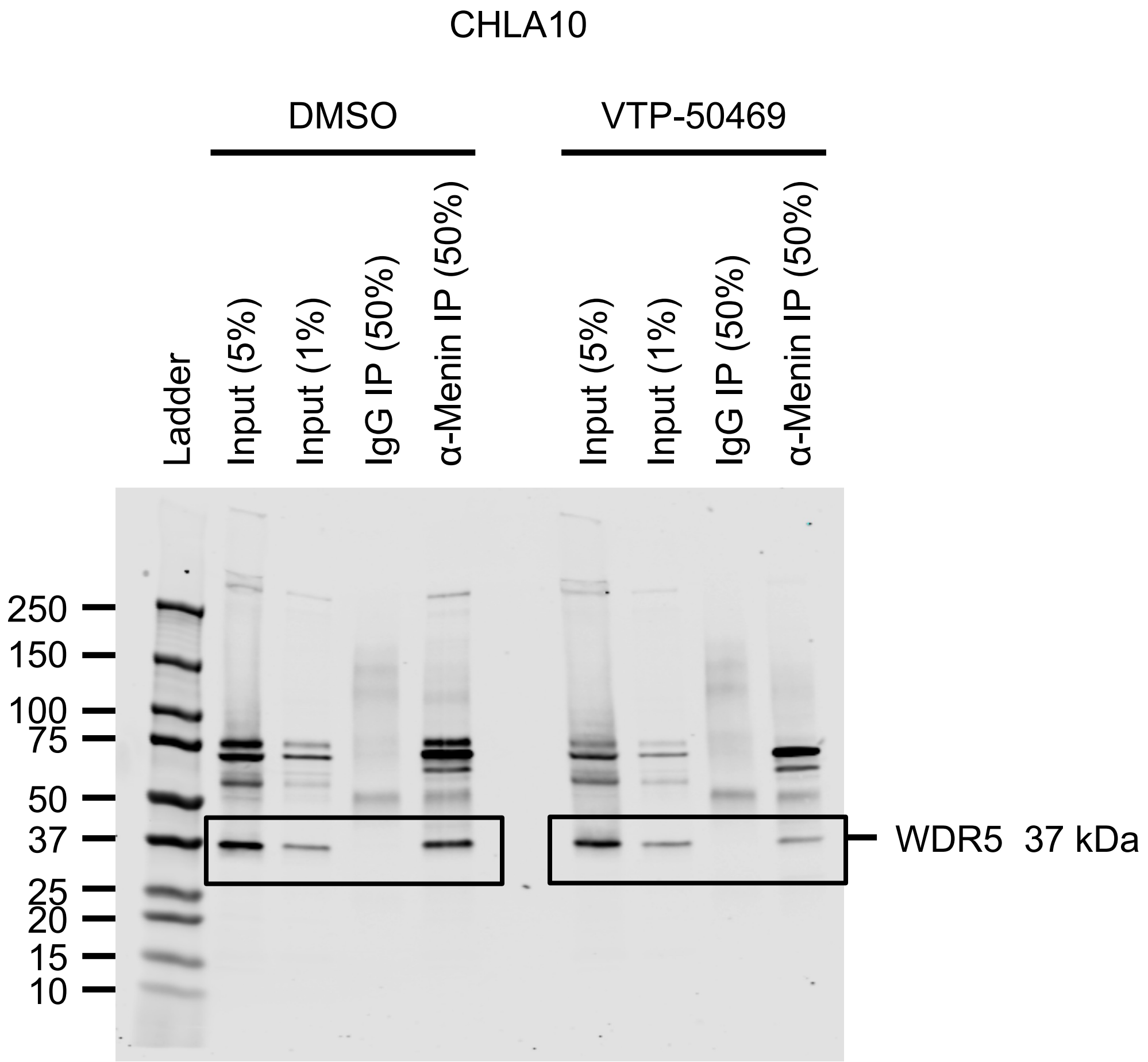

Probed sequentially – α-MYC -> α-Menin -> α-MLL2-C -> α-WDR5

Full unedited blot for Supplemental Figure 2

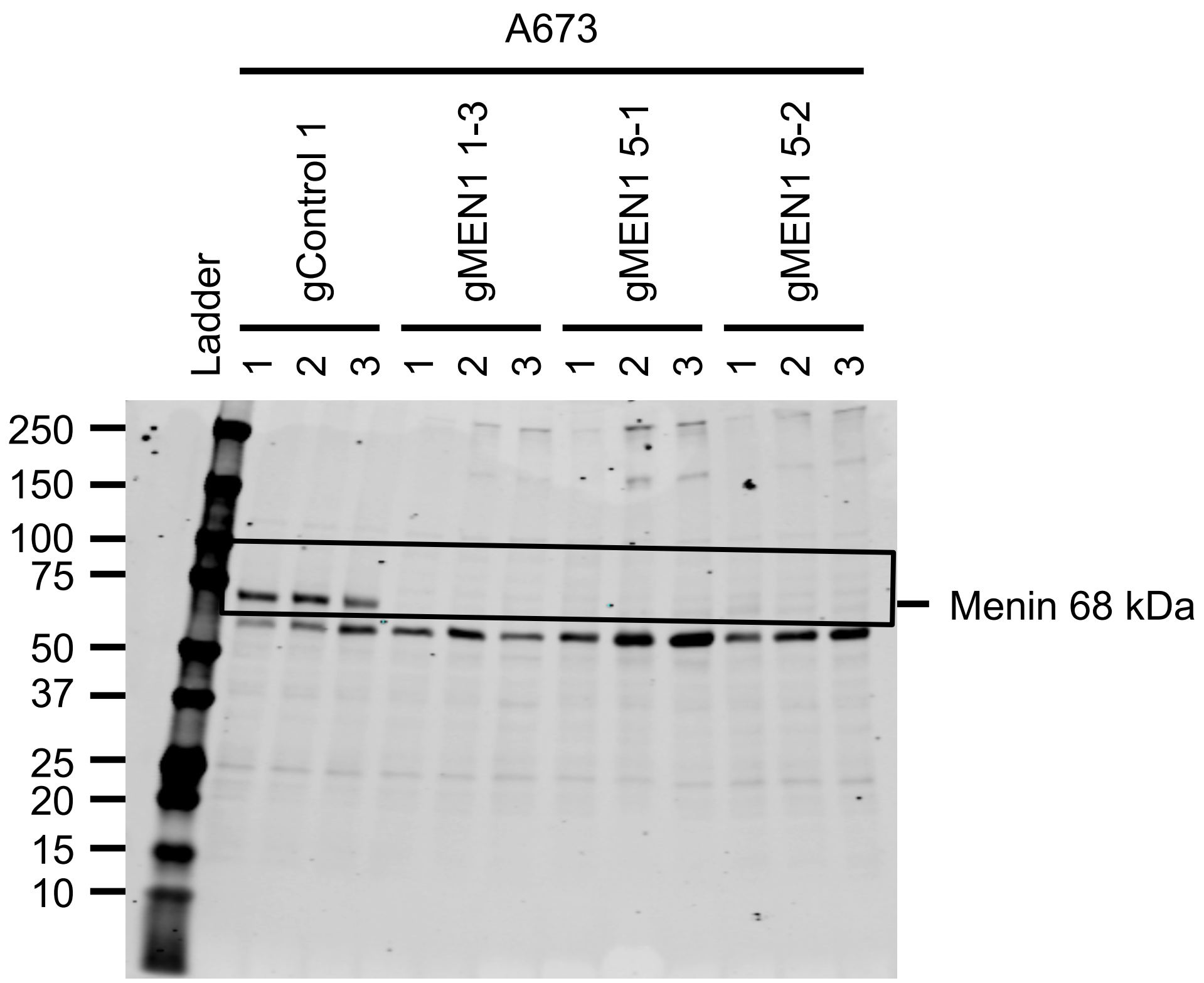

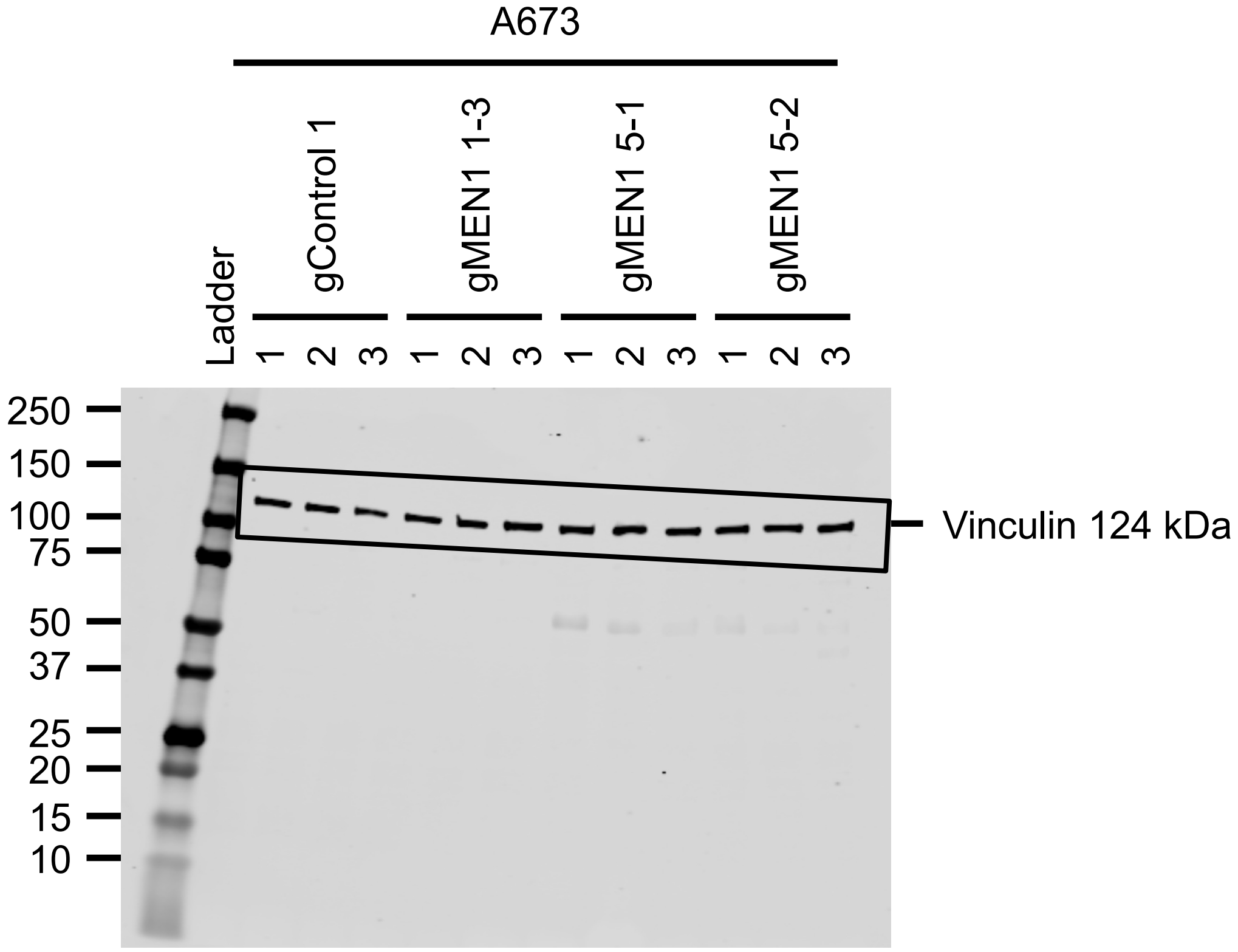

Probed sequentially – α-Menin -> α-Vinculin

Full unedited blot for Supplemental Figure 3

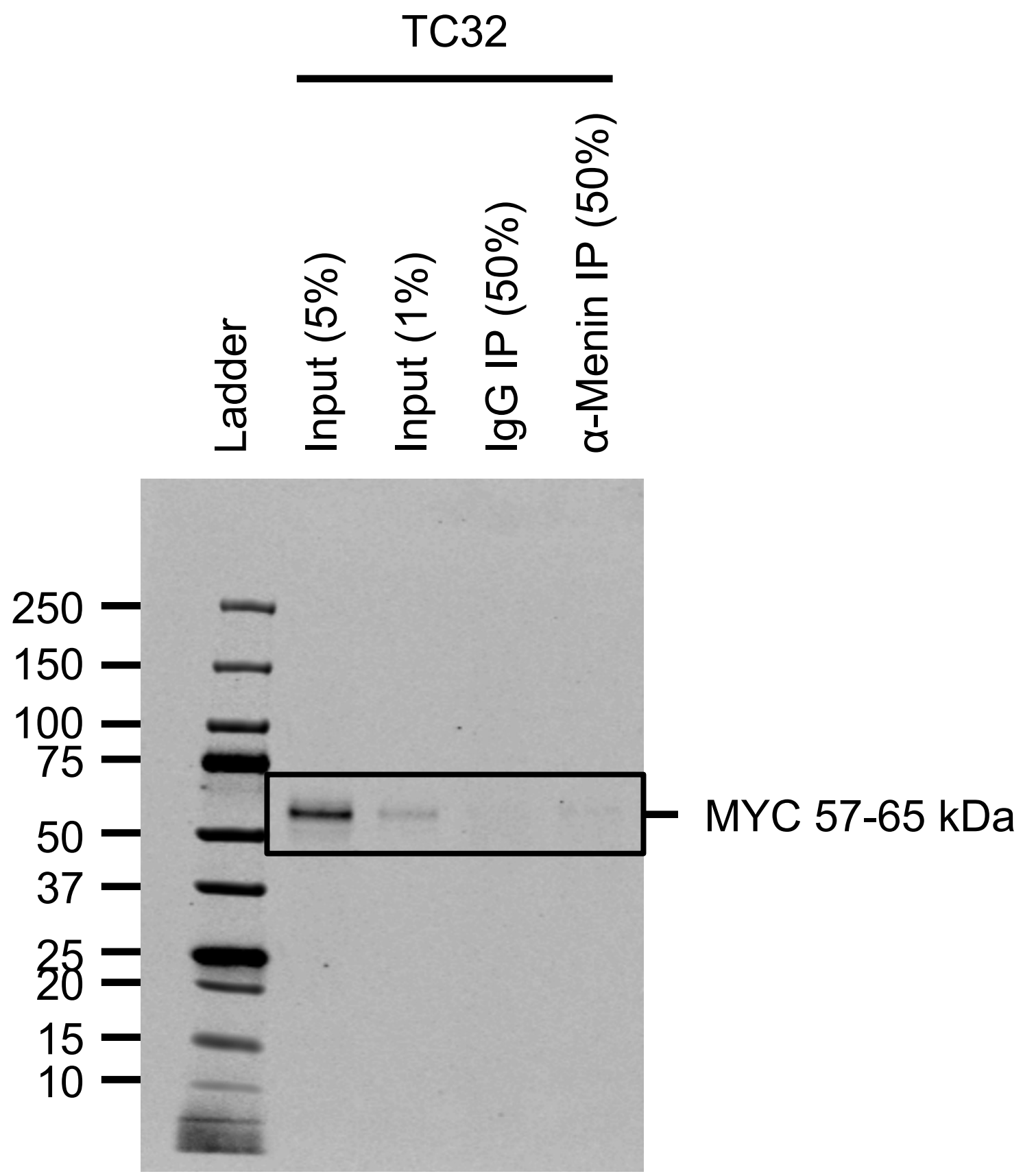

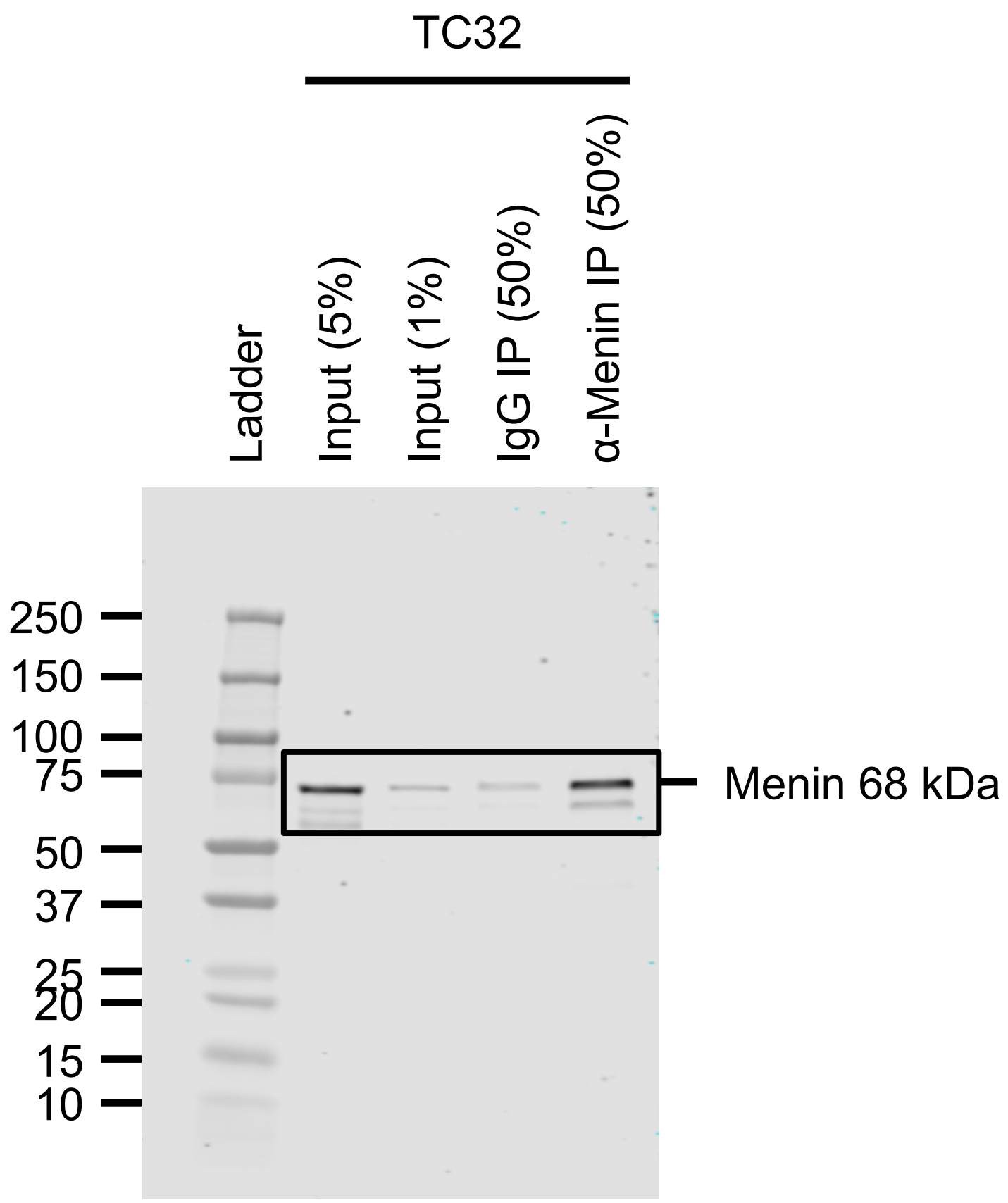

Probed sequentially – α-MYC -> α-Menin

Full unedited blot for Supplemental Figure 3

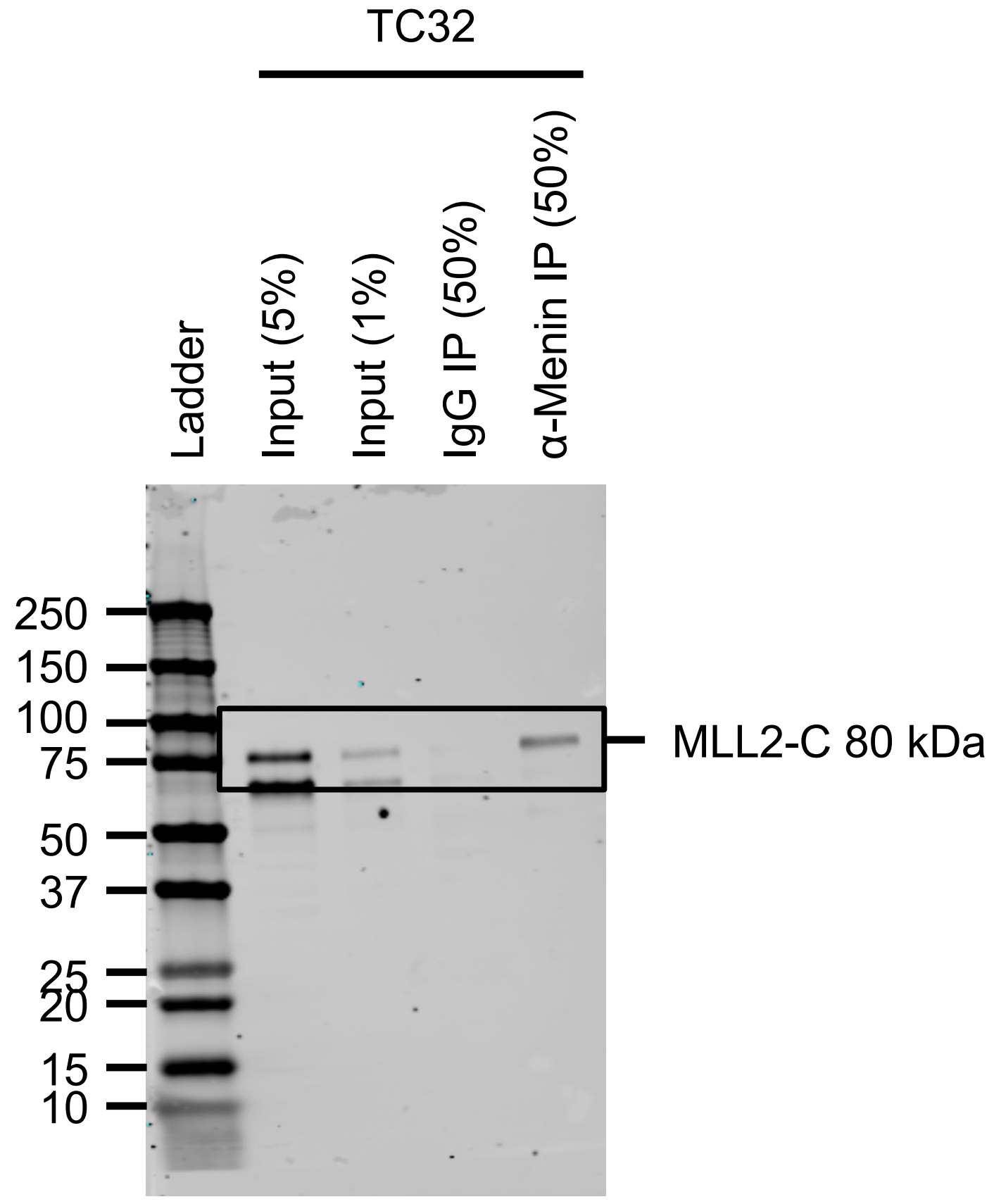

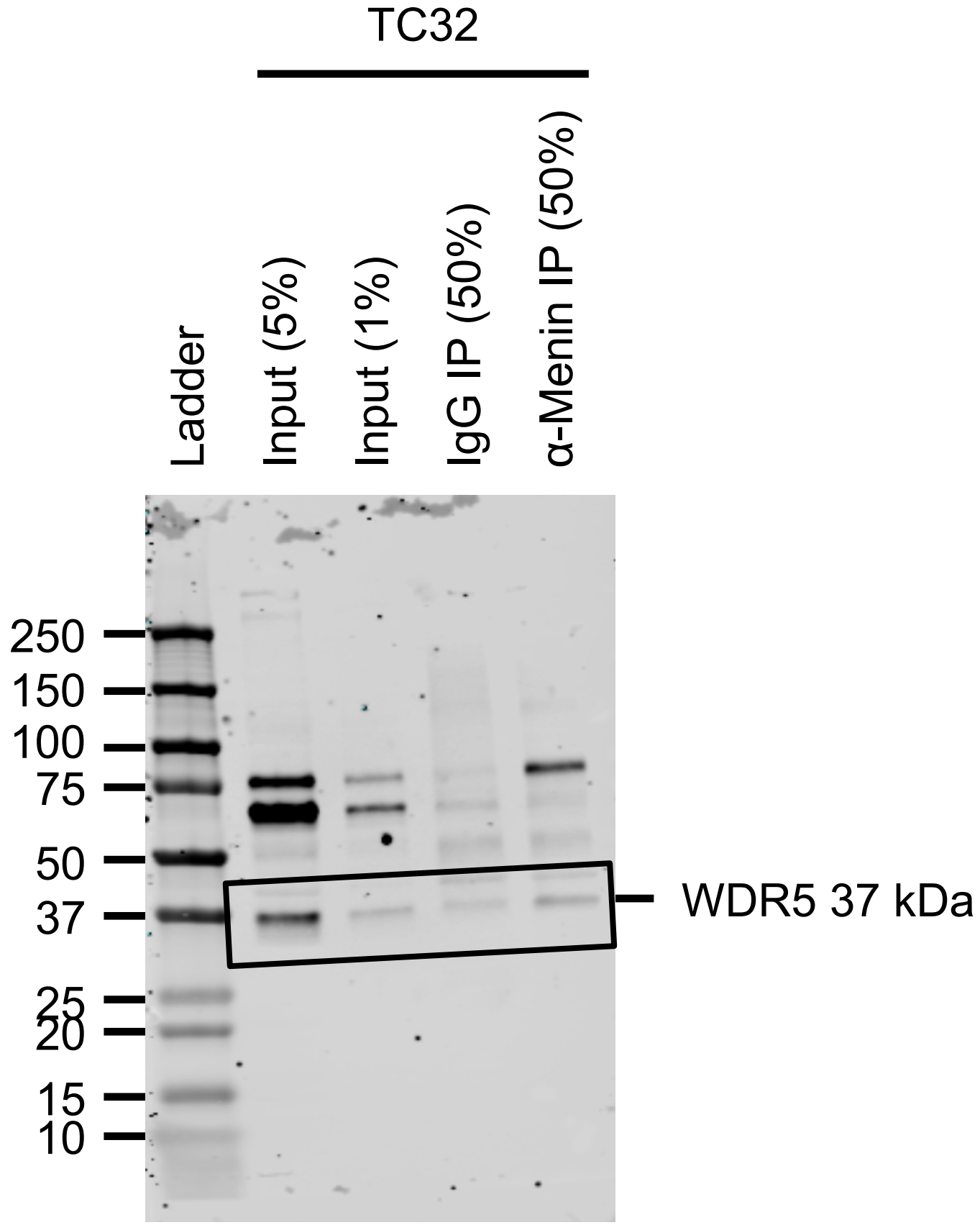

Probed sequentially – α-MLL2-C -> α-WDR5

Full unedited blot for Supplemental Figure 4B

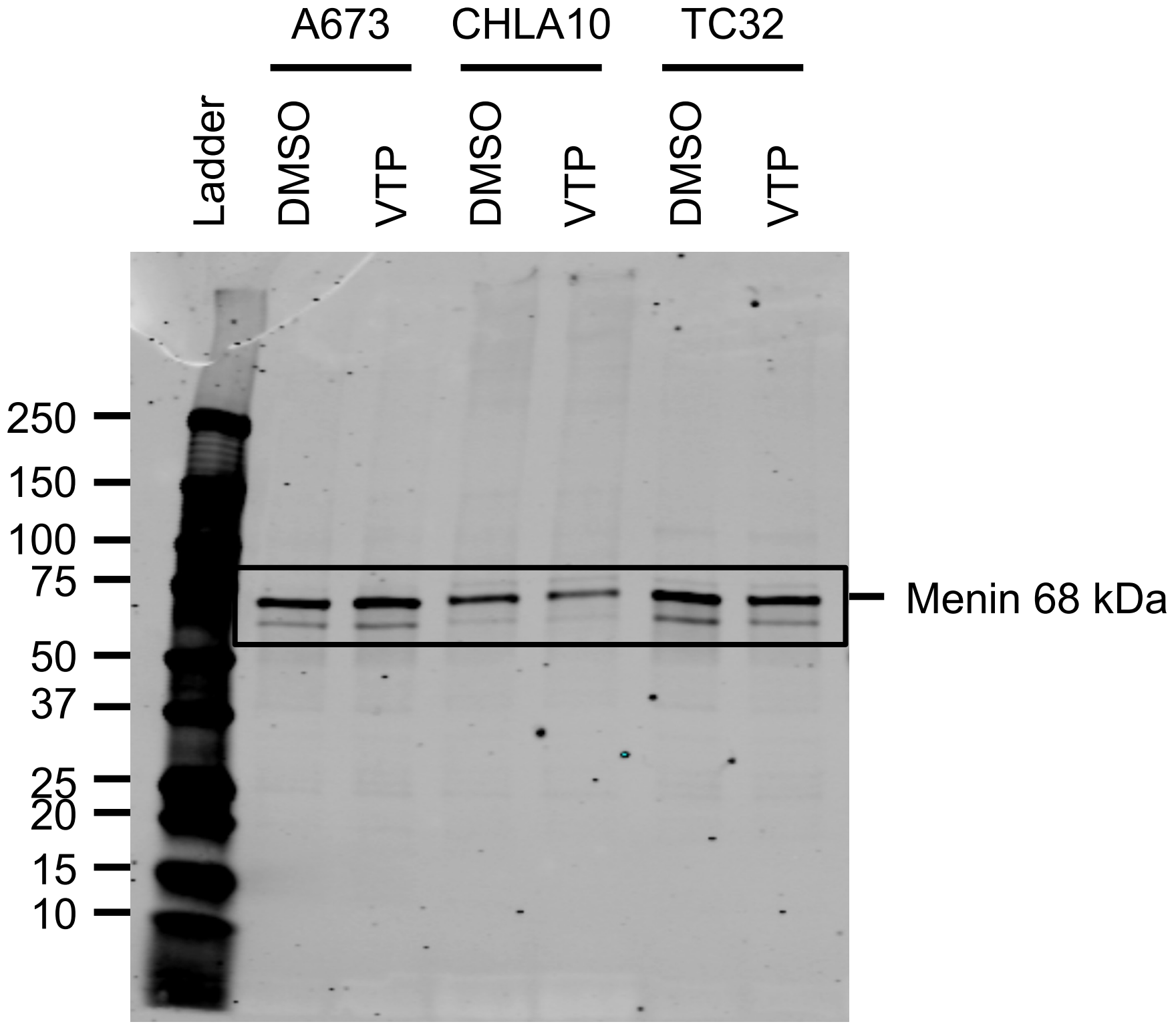

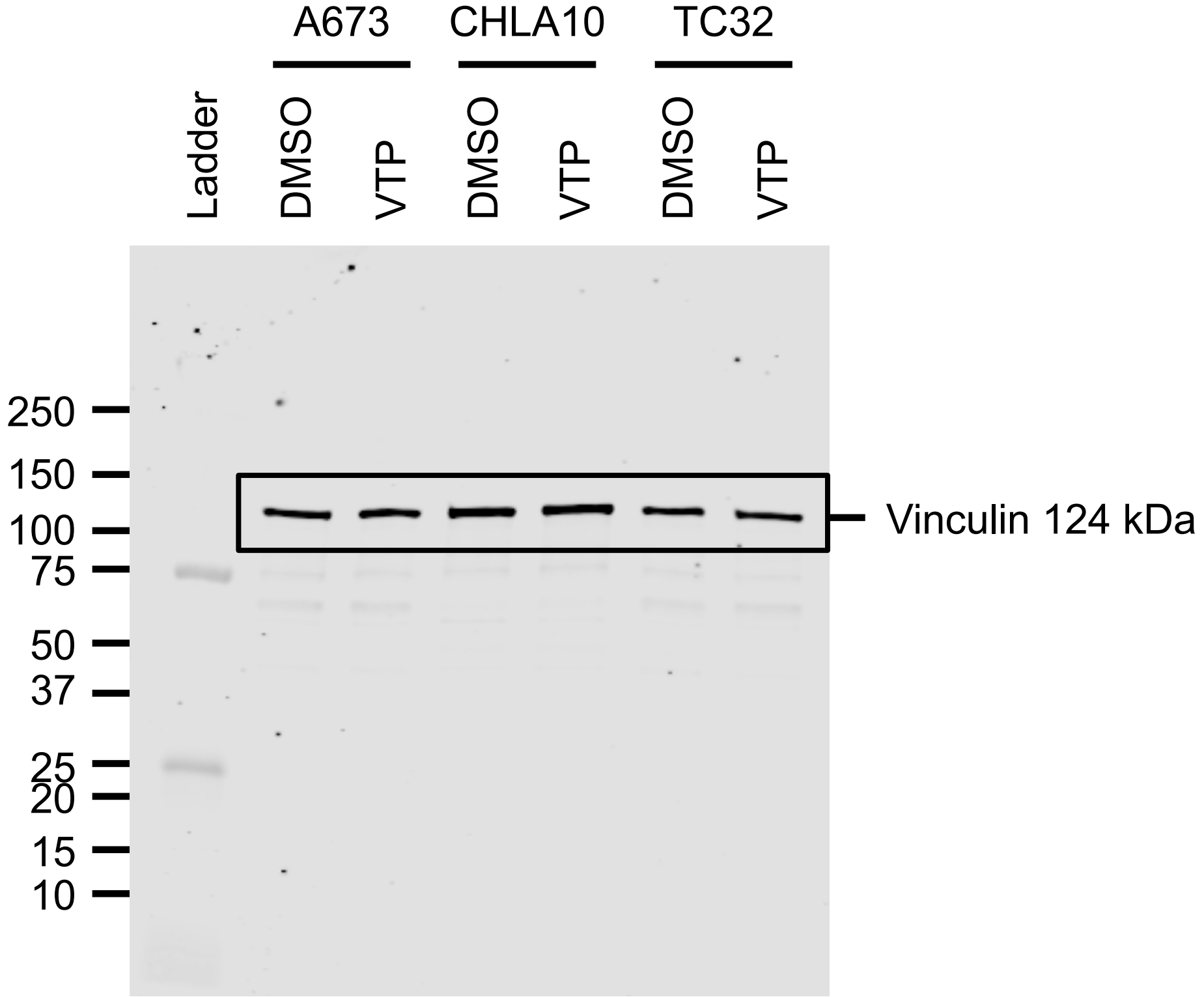

Probed sequentially – α-Menin -> α-Vinculin
